# Polyubiquitination of Transforming Growth Factor β-activated Kinase 1 (TAK1) at Lysine 562 Residue Regulates TLR4-mediated JNK and p38 MAPK Activation

**DOI:** 10.1038/srep12300

**Published:** 2015-07-20

**Authors:** I-Ting Chen, Pang-Hung Hsu, Wan-Ching Hsu, Nien-Jung Chen, Ping-Hui Tseng

**Affiliations:** 1 Institute of Biochemistry and Molecular Biology, School of Life Sciences, National Yang-Ming University, Taipei 11221, Taiwan (ROC); 2 Institute of Bioscience and Biotechnology, College of Life Sciences, National Taiwan Ocean University, Keelung20224, Taiwan (ROC); 3 Institute of Microbiology and Immunology, School of Life Sciences, National Yang-Ming University, Taipei 11221, Taiwan (ROC); 4 Infection and Immunity Research Center, National Yang-Ming University, Taipei 11221, Taiwan (ROC)

## Abstract

Toll-like receptor 4 (TLR4) plays an important role in innate immunity by eliciting inflammation. Upon receptor engagement, transforming growth factor β-activated kinase 1 (TAK1) is an essential mediator that transmits a signal from the receptor to downstream effectors, IκB kinase (IKK) and the mitogen-activated protein kinases (MAPKs), which control the production of inflammatory cytokines. However, the association between phosphorylation and ubiquitination of TAK1 is not yet clear. Here, we examined the crosstalk between phosphorylation and polyubiquitination of TAK1 and further investigated the mechanism of distinct activation of MAPKs and IKK. Inhibition of TAK1 phosphorylation enhanced Lys63-linked polyubiquitination of TAK1. Conversely, ubiquitin modification was counteracted by phospho-mimic TAK1 mutant, T(184,187)D. Moreover, using LC-MS analysis, Lys562 of TAK1 was identified as a novel Lys63-linked ubiquitination site and as the key residue in the feedback regulation. Mutation of Lys562 of TAK1 leads to a decrease in TAK1 phosphorylation and specific inhibition of the MAPK pathway, but has no effect on formation of the TAK1-containing complex. Our findings demonstrate a feedback loop for phosphorylation and ubiquitination of TAK1, indicating a dynamic regulation between TAK1 polyubiquitiantion and phosphorylated activation, and the molecular mechanism by which IKK and MAPKs are differentially activated in the TLR4 pathway.

Transforming growth factor β-activated kinase 1 (TAK1 or MAP3K7), a member of the mitogen-activated protein kinase kinase kinase (MAP3K) family, was first identified in the TGF-beta signaling pathway^1^, and subsequently found to be involved in a variety of inflammatory and immune signaling pathways mediated by toll-like receptors (TLRs), interleukin 1 (IL-1) receptor and the tumor necrosis factor (TNF) receptor family^2^. TAK1, therefore, functions as a crucial regulator of innate and adaptive immunity, cell death, and differentiation.

The IkappaB kinase (IKK)-nuclear factor-κB (NF-κB) pathway and MAPK cascades, such as p38, c-Jun N-terminal kinase (JNK), and extracellular signal-regulated kinase (ERK) are two major effectors of TAK1. TAK1 functions as an essential and positive regulator of NF-κB and MAPK signaling in macrophages, fibroblasts and B cells, but serves as a negative regulator in neutrophils, indicating that TAK1 regulates signals in a cell-specific and receptor-specific manner^3^. TAK1-deficient mouse embryonic fibroblasts (MEFs) or B cells lose the response to TLR ligands, IL-1β or TNF, and fail to activate MAPKs and NF-κB^4^. However, *Map3k7* deletion in neutrophils enhances activation of NF-κB and MAPKs after LPS stimulation^5^. The molecular mechanisms underlying the opposing roles of TAK1 in different cell types remain unknown.

TLR4 is the most-studied of the TLRs. In general, TLR4 signaling is mediated by recruitment of adaptor proteins, such as MyD88 and toll-interleukin-1 receptor (TIR) domain-containing adaptor inducing interferon-beta (TRIF), to the cytoplasmic portions of the receptors and classified as either MyD88-dependent or TRIF-dependent^6^,^7^. In MyD88-dependent signaling, a two-stage signaling mechanism is responsible for spatial and temporal separation of MAPK and IKK-NF-κB signaling^8^. Upon TLR4 engagement, a receptor-associated complex containing MyD88, IL-1 receptor-associated kinase (IRAK) 1, IRAK4, TRAF6, TRAF3, cIAP1, cIAP2, TAK1 and IKKγ is formed. Activation of TAK1 and its downstream targets, the MAPKs, requires translocation of the MyD88-associated complex in to the cytoplasm in a manner that is dependent on cIAP1- or cIAP2-mediated TRAF3 degradation. Phosphorylation-dependent activation of TAK1 subsequently activates MAPK kinase (MKK) 4, MKK7 or MKK6, which are upstream kinases of JNK and p38 MAPK^9^,^10^. Notably, although it has been suggested that TRAF6 interaction with TAK1 may be responsible for the activation of IKK and MAPK pathways^11^, IKK activation is independent of degradative TRAF3-regulated complex translocation, implying different mechanisms for IKK and MAPKs activation in TLR4 signaling. Our recent study suggested that IKK activation might depend on TAK1 protein as a platform for complex formation, but not depend on TAK1 activation^8^. In TNF signaling, it has also been reported that TAK1 activates IKKα and IKKβ due to the dissociation of IKKα and IKKβ from TAK1-containing complex^12^.

In addition to translocation to the cytosol, activation of TAK1 is tightly regulated through its binding partners and protein modifications. TAK1 activation requires the formation of complexes with its binding proteins TAK1-binding protein (TAB) 1, TAB2 or TAB3^13^,^14^,^15^. Post-translational modifications of TAK1 via phosphorylation and polyubiquitination have also be proposed to regulate TAK1 kinase activation^3^. The phosphorylation of Thr184 and Thr187 residues located within the TAK1 activation loop is required for TAK1 kinase activity^16^,^17^, and it has been suggested that “unanchored” Lys63-linked polyubiquitin chains synthesized by TRAF6 trigger TAK1 autophosphorylation on Tyr187^18^. Binding of TAK1 with TAB1 activates TAK1 via autophosphorylation at Ser192 within the kinase domain^19^. Phosphorylation at Ser412 in a PKA-dependent manner is required for full activation of TAK1^20^,^21^. As a relatively rapid and transient signaling process, polyubiquitin chains are added to specific lysine residues of TAK1 by TRAF6 or tripartite motif 8 (TRIM8)^11^,^22^, and trigger TAK1 activation. On the other hand, deubiquitinating enzymes, such as CYLD or ubiquitin specific protease 4 (USP4), can remove the ubiquitin chains from TAK1 and suppress the signal activation^23^,^24^. The Lys209 residue of TAK1 has been identified as the site for Lys63-linked ubiquitin chains in response to IL-1beta^25^. Lys63-linked polyubiquitination at the Lys158 residue has been reported in response to IL-1β, TNF, TGFβ or *Helicobacter pylori*, and regulates the kinase activity of TAK1^26^,^27^,^28^,^29^. Lys63-linked ubiquitin modification at the Lys34 residue of TAK1 in response to different inflammatory stimuli has also been demonstrated^30^,^31^. In addition, Lys72 of TAK1 has been reported to be a Lys48-linked ubiquitination site that downregulates TNF-mediated TAK1 signaling^32^. Moreover, proteomic analyses in murine tissues reveal that Lys563 of TAK1 is ubiquitinated^33^. These data demonstrate that TAK1 is modified by ubiquitin chains at multiple sites.

Biochemical and genetic evidence accumulated so far leaves no doubt that TAK1 plays a major role in the activation of NF-kappaB and the MAPKs in the TLR4 pathway, which is critical for innate immunity. However, the molecular mechanisms underlying TAK1-mediated signal activation, in particular the crosstalk between phosphorylation and ubiquitination, still await elucidation. In this study, we examined the role of TAK1 phosphorylation in activating the IKK and MAPK pathways, and in its interplay with polyubiquitination. Using mass spectrometry, we identified the Lys562 residue of TAK1 as a novel Lys63-linked ubiquitination site that forms a regulatory feedback loop with phosphorylation, and demonstrated that the polyubiqutination of TAK1 at Lys562 is critical for the activation of the MAPKs, but not IKKs, in the TLR4 pathway.

## Results

### TAK1, but not phosphorylated-TAK1, is required for IKK activation

**Figure 1:**
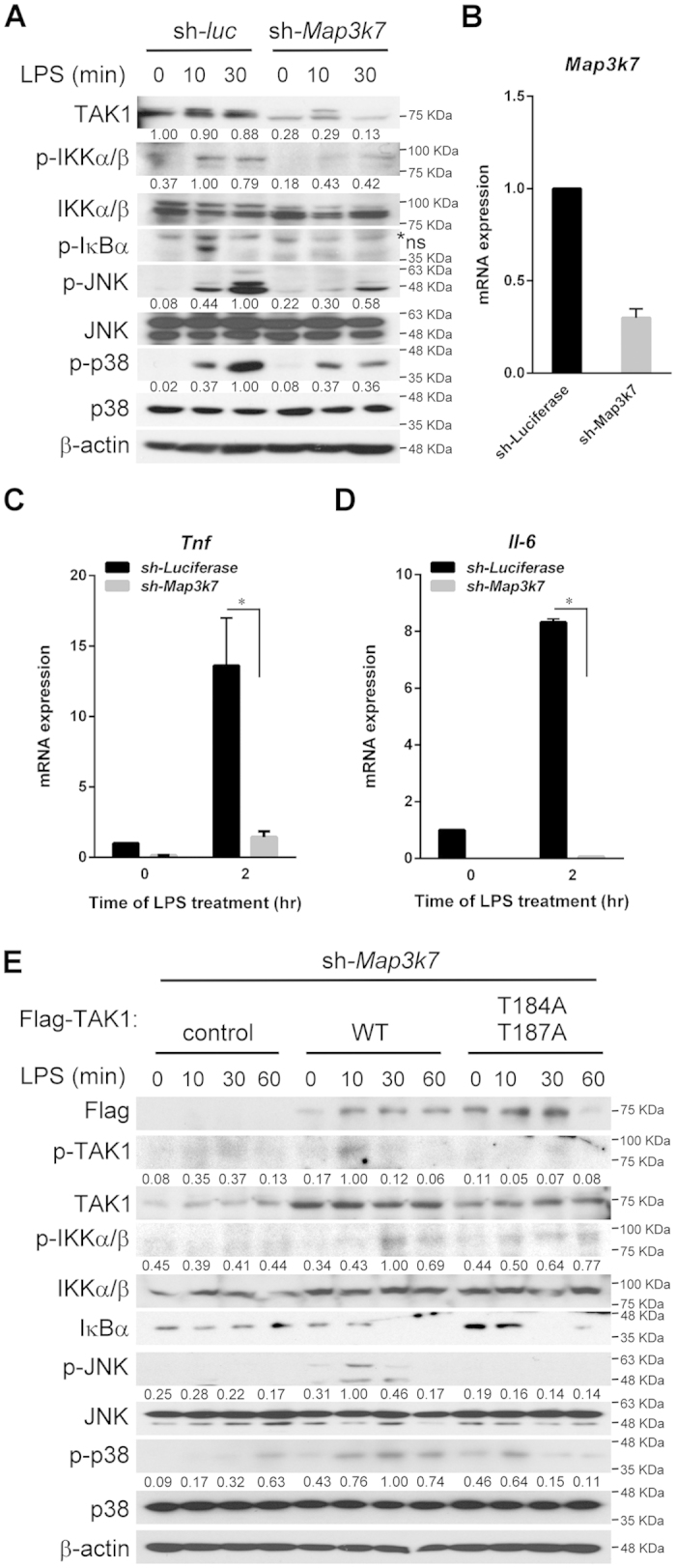
TAK1, but not phosphorylated-TAK1, is required for IKK activation. ***A***, TAK1 is required for IKK and MAPK activation. TAK1 in RAW264.7 cells was knocked down with lentiviral-based shRNA. After treatment with LPS (100 ng/mL) for the indicated times, cells were lysed and subjected to immunoblot analysis. ***B***, mRNA level is decreased in *Map3K7* knocked-down RAW264.7 cells. RAW264.7 cells tranduced with control or shRNA targeting to TAK1 were collected and the total RNA was extracted with TRIzol, reverse transcribed, and analyzed for TAK1 mRNA with Q-PCR. ***C*** and ***D***, TAK1 is required for TLR4-mediated TNF and IL-6 production. Control and TAK1-silenced RAW264.7 cells were stimulated with LPS for 2 h, and total RNA was extracted. After reverse transcription, TNF and IL-6 mRNA were analyzed with Q-PCR. ***E***, phosphorylated-TAK1 is not required for IKK activation. TAK1-silenced RAW264.7 cells were reconstituted with Flag-tagged TAK1 wild-type (WT) or T(184,187)A mutant. The cells were incubated with LPS for the indicated times, lysed and kinase activation was analyzed by immunoblotting. The western blots were quantified with densitometry, the relative amount was calculated and the band with the highest intensity was set as 1. The densitometry data was presented as mean values from three independent experiments. Results of Q-PCR are averages ± SD of three separate experiments (*, *significant difference*).

In agreement with previous reports^4^,^8^, we found that the activation of the MAPKs and IKK was inhibited (Fig. 1A) and the mRNA of proinflammatory cytokines such as TNF and IL6, was diminished in mouse TAK1-knock down RAW264.7 cells stimulated with LPS (Fig. 1C,D). The knockdown efficiency of TAK1 in RAW264.7 cells was about 70% for mRNA level (Fig. 1B) and protein level (Fig. 1A). This indicated that the expression of TAK1 is required for the activation of the MAPKs and IKK, which are critical upstream mediators of proinflammatory cytokine production. Similar results was observed in human TAK1-knock down THP-1 cells (Supplmentary Fig. 1) to confirm that the outcomes of TAK1-silencing were not cell type-specific or due to off-target effect of RNA interference (RNAi). Meanwhile, treatment of TAK1 inhibitor, 5Z-7-oxozeanenol, that inhibits TAK1 kinase activity resulted to the blockage of IKK and MAPKs activation, and the inhibition of cytokine induction, including TNF and IL-6 (Supplementary Figure 2), implying that enzyme activity is critical. However, by reconstituting TAK1-silenced RAW264.7 cells with TAK1 that was mutated in the activation loop (T184 and T187), MAPK signaling was blocked, but the IKK pathway was still intact as compared with wild-type TAK1 (Fig. 1E).

### Lys63-linked polyubiquitination of TAK1 is enhanced by blocking TAK1 phosphorylation

**Figure 2:**
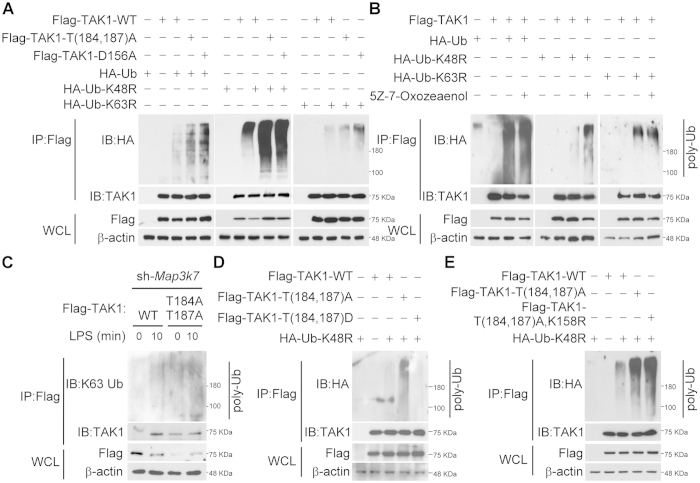
Lys63-linked polyubiquitination of TAK1 is enhanced by blocking TAK1 phosphorylation. ***A***, TAK1 polyubiquitnation is enhanced by blocking TAK1 phosphorylation. Flag-tagged TAK1 wild-type (WT), T(184,187)A, or D156A mutants were co-expressed in HEK293T cells with HA-tagged Ub WT, K48R, or K63R mutants. The cells were lysed, immunoprecipitated with anti-Flag antibody, extensively washed, and gel separated. Polyubiquitination was detected with anti-HA antibody. ***B***, TAK1 polyubiquitination is enhanced by inhibiting TAK1 activation. Flag-tagged WT TAK1 was co-expressed in HEK293T cells with HA-tagged Ub WT, K48R, or K63R mutants. After treatment with TAK1 inhibitor 5Z-7-oxozeanenol (100 nM) for 30 min, the cells were lysed, immunoprecipitated, and ubiquitination was detected with anti-HA antibody. ***C***, in response to LPS, TAK1 polyubiquitnation is enhanced in TAK1 T(184,187)A mutant. TAK1-silenced RAW264.7 cells were reconstituted with Flag-tagged TAK1 wild-type (WT) or T(184,187)A mutant. The cells were incubated with LPS for the indicated times, lysed, immunoprecipitated with anti-Flag antibody, extensively washed, and gel separated. Polyubiquitination was detected with anti-Lys63-linked Ub antibodies. ***D***, non-Lys48-linked polyubiquitination of TAK1 is blocked by TAK1 phosphorylation. Flag-tagged TAK1 wild-type (WT), T(184,187)A, or T(184,187)D mutants were co-expressed in HEK293T cells with HA-tagged K48R Ub mutant. The cells were collected, and analyzed using *in vivo* ubiquitination assay. ***E***, enhancement of TAK1 polyubiquitination induced by TAK1 inhibition is not at the Lys158 residue. Flag-tagged TAK1 wild-type (WT), T(184,187)A, or T(184,187)A, K158R mutants were co-expressed in HEK293T cells with HA-tagged Ub K48R mutant. The cells were collected and analyzed for polyubiquitination. The result are represented from at least three independent experiments.

Lys63-linked polyubiquitination of TAK1 is required for the activation of TAK1 and the downstream IKK and MAPKs pathways^34^. It has also been shown that crosstalk between phosphorylation and ubiquitination occurs at several levels^35^. To further investigate the association between TAK1 phosphorylation and polyubiquitnation, either TAK1 wild-type (WT) or mutants that mutated in the activation loop T(184,187)A or substrate binding site D156A^36^ were overexpressed in HEK293T cells. Surprisingly, polyubiquitination of TAK1 as detected by *in vivo* ubiquitination assay was enhanced when T(184,187)A- or D156A-TAK1 was expressed (Fig. 2A). Treatment with TAK1 inhibitor, 5Z-7-oxozeanenol, produced a similar result (Fig. 2B). The enhancement of polyubiquitination was more significant in the K48R Ub mutant (Fig. 2A,B), suggesting the major mode of accumulated polyubiquitination was non-Lys48-linked. Next, Lys63-linked specific anti-ubiquitin antibody was used to examined the mode of TAK1 polyubiquitination. In line with the result from HEK293T cells, Lys63-linked ubiquitination was enhanced when TAK1 mutant, T(184,187)A, was overexpressed in RAW264.7 cells in response to LPS stimulation (Fig. 2C). Besides, enhancement of Lys63-linked polyubiquitination in response to LPS could also be observed in RAW264.7 or THP-1 cells pretreated with TAK1 inhibitor, 5Z-7-oxozeanenol (Supplementary Figure 3). To further confirm this observation, HEK293T cells were expressed with phospho-mimic TAK1 mutant, T(184,187)D. *In vivo* ubiquitiantion assay showed that non-Lys48-linked polyubiquitination of TAK1 was inhibited (Fig. 2D). However, phosphor-mimic T(184,187)D, but not T(184,187)A), TAK1 mutant was able to activated MAPKs-mediated AP-1 transcription activity with AP-1 reporter assay (Supplementary Figure 4). Together, these results suggested a checkpoint for polyubiquitination and phosphorylation that inhibiting TAK1 activation might lead to accumulation of Lys63-linked TAK1 polyubiquitnation, while TAK1 phosphorylated activation might transmit signal to stop further ubiquitination.

It has been reported that Lys158 residue of TAK1 is a Lys63-linked ubiquitination site, and is critical for TAK activation^28^. Therefore, we next examined whether the polyubiquitiantion is at Lys158 residue of TAK1. Overexpression of TAK1 mutant, T(184,187)A, K158R, in HEK293T cells did not block the increase in polyubiquitination (Fig. 2E), indicating that Lys158 is not the site of modification resulted from inhibiting TAK1 activation.

### Lys562 residue of TAK1 is a Lys63-linked ubiquitination site

**Figure 3:**
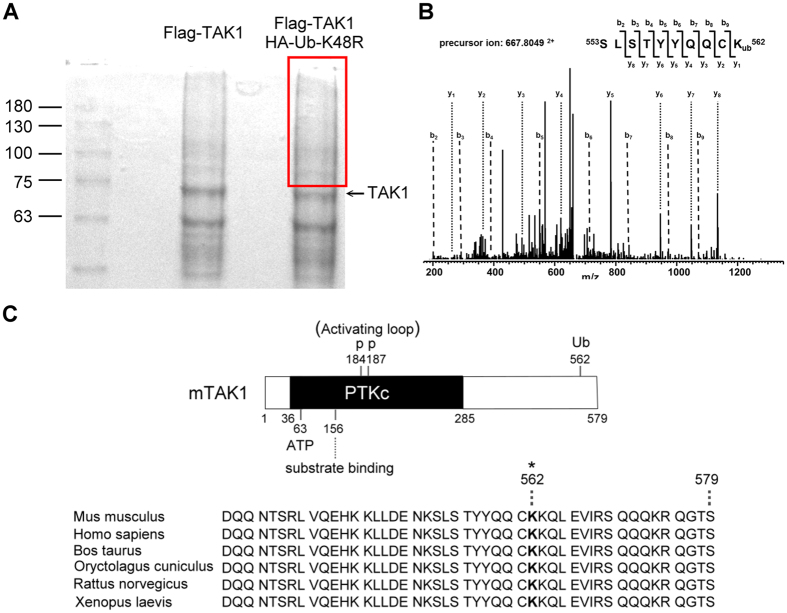
Lys562 residue of TAK1 is an ubiquitination site. ***A***, Coomassie blue stained gel indicates the band with TAK1 polyubiquitination. Flag-tagged TAK1 with or without HA-tagged ubiquitin were expressed in HEK293T cells. Cells were lysed, immunopricipitated, gel-separated, and stained with Coomassie blue. The square area indicated the presence of ubiquinated-TAK1. ***B***, Lys562 residue of TAK1 was identified as an ubiquitination site by mass spectrometry. The protein sample with ubiquitinated TAK1 was recovered from the gel, enzyme digested, and subjected to mass analysis. Representative MS/MS spectra of peptides demonstrated ubiquitination at Lys562 of TAK1. Peaks matching expected b and y ions are labeled. ***C***, Upper panel: schematic structure of TAK1, indicating functional domains and the new site identified in this study. Lower panel: alignment of part of the C-terminal of TAK1. The Lys562 residue is indicated by an asterisk.

Since the enhanced polyubiquitination caused by inhibition of TAK1 activation was not at Lys158, it is very likely that TAK1 contains multiple ubiquitination sites in response to signal activation. Flag-tagged TAK1 and ubiquitin were co-expressed in HEK293T cells to force ubiquitination. After immunoprecipitating ubiquitinated TAK1 with anti-Flag antibody, the sample was separated by SDS-PAGE and visualized by Coomassie blue (Fig. 3A). In order to analyze ubiquitinated TAK1 by MS, the gel region containing ubiquitin-modified protein was excised, digested with trypsin, and analyzed by LC-MS/MS. A database search of the MS/MS spectra revealed that TAK1 was the predominant protein identified in the samples. Representative spectra that demonstrate that the Lys562 residue of TAK1 was identified to be modified with ubiquitin with 60% sequence coverage are shown in Fig. 3B. Comparison of the C-terminal protein sequence of TAK1 in different species showed that the Lys562 residue was highly conserved (Fig. 3C).

**Figure 4:**
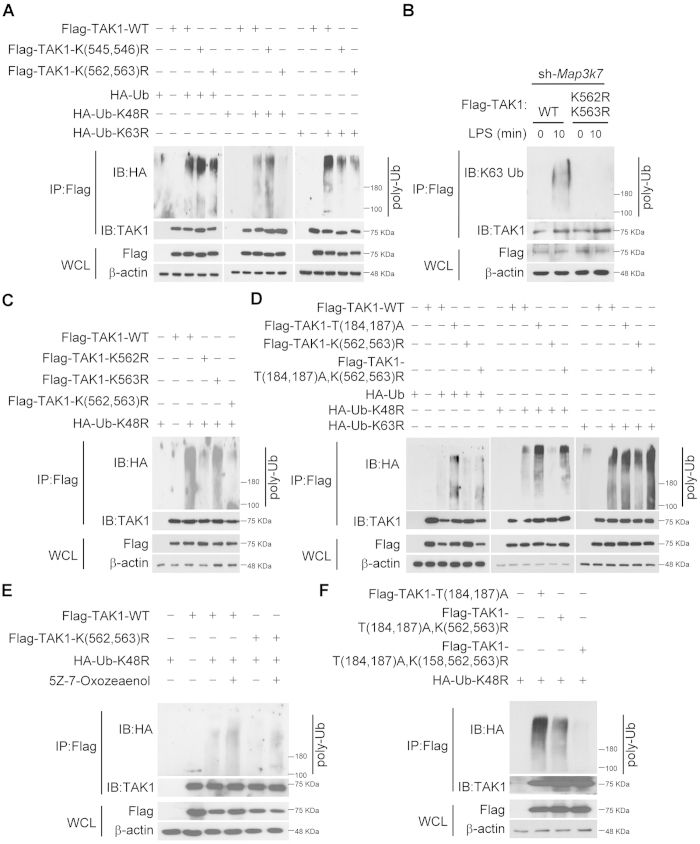
Lys562 residue of TAK1 is a Lys63-linked ubiquitination site. ***A***, Lys562 or Lys563 residue of TAK1 undergoes non Lys48-linked polyubiquitination. Flag-tagged TAK1 wild-type (WT), K(545,546)R, or K(562,563)R mutants were co-expressed in HEK293T cells with HA-tagged Ub WT, K48R, or K63R mutants, and analyzed for polyubiquitination as described above. ***B***, in response to LPS, Lys562 or Lys563 residue of TAK1 undergoes Lys63-linked polyubiquitination. TAK1-silenced RAW264.7 cells were reconstituted with Flag-tagged TAK1 wild-type (WT) or K(562,563)R mutant. The cells were incubated with LPS for the indicated times, lysed, immunoprecipitated, and detected polyubiquitination with anti-Lys63-linked Ub antibodies. ***C***, Lys562 residue of TAK1 undergoes Lys63-linked polyubiquitination. Flag-tagged TAK1 wild-type (WT), K562R, K563R, or K(562,563)R mutants were co-expressed in HEK293T cells with HA-tagged K48R mutants. Lysates of the cells were analyzed for polyubiquitination. ***D***, TAK1 polyubiquitnation enhanced by blocking TAK1 phosphorylation is located at the Lys562 residue. Flag-tagged TAK1 wild-type (WT), T(184,187)A, K(562,563)R, or T(184,187)A, K(562,563)R mutants were co-expressed in HEK293T cells with HA-tagged Ub WT, K48R, or K63R mutants. The cells were collected and analyzed for polyubiquitnation. ***E***, TAK1 polyubiquitnation enhanced by TAK1 inhibition is located at the Lys562 residue. Flag-tagged TAK1 wild-type (WT), or K(562,563)R mutant was co-expressed in HEK293T cells with HA-tagged Ub K48R mutant. After treatment with TAK1 inhibitor 5Z-7-oxozeanenol for 30 min, the cells were collected and analyzed for polyubiquitnation. ***F***, polyubiquitination at Lys158 residue of TAK1 is independent to Lys562 residue. Flag-tagged TAK1 T(184,187)A, T(184,187)A, K(562,563)R, or T(184,187)A, K(158,562,563)R mutants were co-expressed in HEK293T cells with HA-tagged Ub K48R mutant. The cells were collected and analyzed for polyubiquitnation. The result are represented from at least three independent experiments.

The result from mass analysis that Lys562 residue of TAK1 as an ubiquitination site was further confirmed with *in vivo* ubiquitination assay. Because TAK1 residue 563 next to Lys562 is also a lysine and also reported as an ubiquitination site^33^, K(562,563)R mutant was used to prevent non-specific modification. Mutants in the nearby lysine residues Lys545 and 546, which are conserved across various species (Fig. 3C) were used as negative controls. TAK1 WT, K(562,563)R and K(545,546)R mutants were overexpressed in HEK293T cells, respectively. Polyubiquitination was significantly decreased in K(562,563)R mutant, in particular when K48R ubiquitin was co-transfected (Fig. 4A). This result indicated that the Lys562 or Lys563 residue of TAK1 is the ubiquitination site and the modification is mainly non-Lys48-linked ubiquitination. To confirm the mode of polyubiquitination, Lys63-linked ubiquitination was diminished in TAK1-silenced RAW264.7 or THP-1 cells reconstituted with TAK1 K(562,563)R mutant in response to LPS stimulation (Fig. 4B and Supplementary Figure 5A). Furthermore, the role of TAK1 Lys562 or Lys563 residue as an ubiquitination site was investigated. With *in vitro* ubiquitination assay, TRAF6-mediated Lys63-linked polyubiquitination of TAK1 was detected in WT and K562R, but not in K(562,563)R mutant (Supplementary Figure 5B and C). Although the polyubiquitination of K562R TAK1 was still modified with polyubiquitination, it might be due to non-specific reaction on nearby Lys563 residue. *In vivo* ubiquitination assay was used for further examination. WT, K562R, K563R and K(562,563)R mutants were overexpressed in HEK293T cells, respectively. Polyubiquitination was significantly decreased in K562R and K(562,563)R, but not in K563R mutant (Fig. 4C). This result supported the finding from mass analysis that the Lys562 residue of TAK1 is Lys63-linked ubiquitination site. Additionally, in response to Pam3CSK4 stimulation, Lys63-linked ubiquitination was diminished in TAK1-silenced RAW264.7 reconstituted with TAK1 K(562,563)R mutant (Supplementary Figure 5D), indicating the modification at Lys562 residue is not TLR4-specific.

Next, whether the Lys562 residue is the site of polyubiquitination when TAK1 activation is blocked was examined. Again, K(562,563)R mutant showed decreased non-Lys48-linked polyubiquitination comparing with WT, and T(184,187)A mutant enhanced non-Lys48-linked polyubiquitination. When TAK1 mutant T(184,187)A, K(562,563)R was co-expressed with ubiquitin WT or K48R mutant in HEK293T cells, the enhancement in polyubiquitination was blocked (Fig. 4D). However, when TAK1 mutant T(184,187)A, K(562,563)R was co-expressed with K63R Ub mutant, the enhancement in polyubiquitination was not inhibited, indicating Lys562 residue of TAK1 might not be involved in the increase of Lys48-linked ubiquitination with TAK1 inhibition, and Lys72 residue of TAK1 might be the site responding to the Lys48-linked ubiquitination^32^. Meanwhile, the accumulation of non-Lys48-linked polyubiquitination resulted from treatment of TAK1 inhibitor was decreased in HEK293T cells expressed with K(562,563)R TAK1 mutant and K48R Ub mutant (Fig. 4E). These data suggest that the Lys562 residue is the site of over-modification with Lys63-linked ubiquitin when TAK1 activation is inhibited.

Since non-Lys48-linked polyubiquitination was still able to be detected in K562R or K(562,563)R mutated TAK1 as shown in Fig. 4C,D, the remaining ubiquitination might by at Lys158 residue. With triple mutation of TAK1 K(158,562,563)R, polyubiquitination enhanced by T(184,187)A mutant can be further blocked comparing to TAK1 K(562,563)R, indicating that polyubiquitination at Lys158 residue of TAK1 is independent to Lys562 residue (Fig. 4F).

### Polyubiquitination of TAK1 at Lys562 is required for the activation of TAK1 and the MAPKs, but not IKK upon LPS stimulation

**Figure 5:**
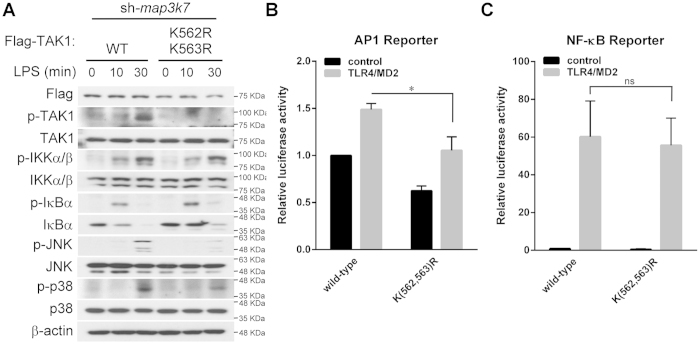
Polyubiquitination of TAK1 at Lys562 is required for the activation of TAK1 and MAPK, but not IKK. ***A***, mutation in Lys562 residue of TAK1 in IKK, in response to LPS. TAK1-silenced RAW264.7 cells were reconstituted with Flag-tagged TAK1 wild-type (WT) or K(562,563)R mutant. The cells were incubated with LPS for the indicated times, lysed and phosphorylated proteins were analyzed with immunoblotting (n = 3). ***B*** and ***C***, expression of the TAK1 K(562,563)R mutant lead to declined TLR4-mediated AP-1 reporter activity, but had no effect on NF-κB reporter activity. Flag-tagged TAK1 wild-type (WT) or K(562,563)R mutant, Renilla-Luc plasmid and either AP-1 or NF-κB reporter were co-expressed in HEK293T cells with or without TLR4-MD2 for 24 h. Relative luciferase activity was measured and normalized with the Renilla activity. Results are averages ± SD of three separate experiments (*, *significant difference*).

The results above suggested that Lys562 of TAK1 is a novel non-Lys48-linked ubiquitination site. Next, to dissect the role of Lys562 in TAK1-mediated signaling TAK1-silenced RAW264.7 cells were reconstituted with TAK1 WT or K(562,563)R mutant, and TAK1-mediated signal activation, including IKK, p38 and JNK, in TLR4 pathway was analyzed. Unlike the Lys158 site of TAK1 that has been reported to be required for TAK1-mediated IKK and MAPK activation in response to TNF stimulation^28^, mutation in Lys562 residue of TAK1 led to impairment in TAK activation, and downstream MAPKs, but not IKK signaling in response to LPS stimulation (Fig. 5A and Supplementary Figure 6A). To confirm the activation of the MAPKs and IKK pathway, AP-1 and NF-κB luciferase reporter assay were performed. TAK1 knock-down HEK293 cells were reconstituted with WT and K(562,563)R mutant TAK1 with or without overexpression of TLR4/MD2, and the result showed decreased AP-1 transcription activity (Fig. 5B), but no significant difference in NF-κB transcription activity (Fig. 5C). Furthermore, similar results were observed in RAW264.7 cells stimulated with IL-1 and TNF (Supplementary Figure 6B and C). Also, the AP-1 reporter assay with TAK1 K562R, or K(562,563)R mutants were decreased when TLR2 was overexpressed (Supplementary Figure 6D). These data suggest that in response to various ligand stimulation, ubiquitination of TAK1 on Lys562 is important for TAK1 activation, and, thus, critical for MAPK, but not IKK, activation.

### Mutation at Lys562 of TAK1 does not disrupt the interaction of TAK1 with TRAF6 or Tab1

**Figure 6:**
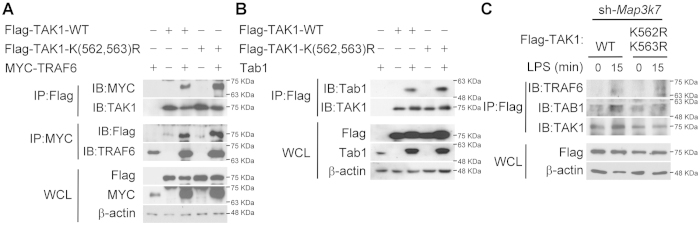
Mutation in Lys562 residue of TAK1 does not disrupt the interaction of TAK1 with TRAF6 or TAB1. ***A***, Mutation in Lys562 residue of TAK1 does not disrupt the interaction of TAK1 with TRAF6. Flag-tagged TAK1 wild-type (WT) or K(562,563)R mutant were co-expressed in HEK293T cells with myc-tagged TRAF6. The cells were lysed and immunoprecipitated with anti-Flag or anti-myc antibody. After gel separation, TAK1-containing complex was detected with the indicated antibodies. ***B***, Mutation in Lys562 residue of TAK1 does not interrupt the interaction of TAK1 with TAB1. Flag-tagged TAK1 wild-type (WT), or K(562,563)R mutant were co-expressed in HEK293T cells with TAB1. The cells were lysed, immunoprecipitated, gel separated, and detected with the indicated antibodies. ***C***, in response to LPS, the interaction of TAK1 with TRAF6 and TAB1 is not disrupted by TAK1 with mutation of Lys562 residue. TAK1-silenced RAW264.7 cells were reconstituted with Flag-tagged TAK1 wild-type (WT) or K(562,563)A mutant. The cells were incubated with LPS for the indicated times, lysed, immunoprecipitated, gel separated and detected with the indicated antibodies. The result are represented from at least three independent experiments

We previously demonstrated that MyD88-dependent TAK1 signaling in TLR4 proceeds via a two stage mechanism that involves receptor-induced assembly of a multiprotein complex, containing MyD88, TRAF6, IKKγ/NEMO, TAK1 and TRAF3, and degradative TRAF3-mediated translocation of the MyD88-associated signaling complex to the cytosol^8^. In addition, TAK1 is constitutively associated with TAB1 and activated by autophosphorylation^19^. Also in a previous report^26^, polyubiquitination of TAK1 at Lys158 residue was found critical for binding with IKKγ/NEMO. Therefore, next, the role of the ubiquitinated Lys562 residue in complex formation was examined. To investigate the interaction between TAK1 and TRAF6, Flag-tagged TAK1 WT, T(184,187)A, or K(562,563)R mutants and Myc-tagged TRAF6 were co-expressed in HEK293T cells. After immunoprecipitating, the TAK1-containing complex was detected. The result showed that the binding between TAK1 and TRAF6 is independent of phosphorylation in the activation loop or ubiquitination at Lys562 (Supplementary Figure 7 and Fig. 6A). To explore the association between TAK1 and TAB1, HEK293T cells were co-expressed with Flag-tagged TAK1 WT, or K(562,563)R mutants and TAB1. After immunoprecipitating TAK1 with anti-Flag antibody, the TAK1-TAB1 complex was detected with anti-Tab1 antibody. The result indicated that the polyubiquitination at Lys562 residue located within the TAB2-binding domain is not involved in the interaction between TAK1 and TAB1 (Fig. 6B). By reconstituting TAK1-silenced RAW264.7 cells with TAK1 WT or K(562,563)R, the amount of coprecipitated TRAF6 or TAB1 in TAK1-containing complex was not changed in response to LPS engagement (Fig. 6C), suggesting that Lys562 of TAK1 did not interfere with interaction of TAK1 with TRAF6 or TAB1.

### Inhibiting polyubiquitination of TAK1 at Lys562 residue leads to a decline in cytokine production upon LPS stimulation

**Figure 7:**
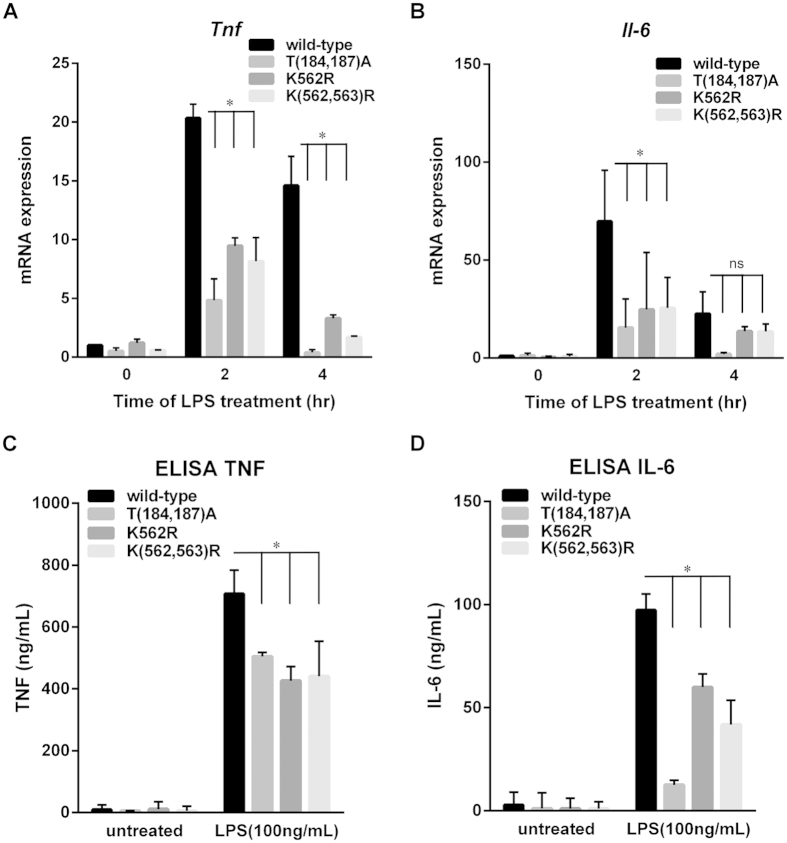
Inhibition of polyubiquitination of TAK1 at Lys562 residue leads to a decline in cytokine production. ***A*** and ***B***, TAK1 is required for TLR4-mediated induction of TNF and IL-6 mRNA. TAK1-silenced RAW264.7 cells were reconstituted with Flag-tagged TAK1 wild-type (WT), T(184,187)A, K562R or K(562,563)R mutant. After treatment with LPS for indicated times, the cells were collected and the total RNA was extracted, reverse transcribed, and analyzed for TNF and IL-6 mRNA with Q-PCR. ***C*** and ***D***, TAK1 is required for TLR4-mediated TNF and IL-6 production. TAK1-silencing RAW264.7 cells were reconstituted with Flag-tagged TAK1 wild-type (WT), T(184,187)A, K562R or K(562,563)R mutant. After incubating with LPS for 24 h, the medium was collected and analyzed for TNF and IL-6 level with ELISA. Results are averages ± SD of three separate experiments (*, *significant difference*).

TAK1 mediates IKK and MAPK activation which in turn induce inflammatory cytokines, such as TNF and IL-6. Next, the role of Lys562 in TAK1-mediated gene expression was investigated. TAK1-silenced RAW264.7 cells were reconstituted with either TAK1 WT, T(184,187)A, K562R, or K(562,563)R mutants, and TAK1-induced mRNA expression, including that of TNF and IL6, in response to LPS stimulation was analyzed with RT-qPCR (Fig. 7A,B). It was shown previously that Lys562 residue of TAK1 is critical for MAPKs activation. Meanwhile, MAPKs pathway has been indicated to regulate inflammatory cytokines, including TNF and IL-6 through multiple mechanisms^37^,^38^,^39^. Therefore, it was no surprise that the mRNA level of TNF and IL-6 was decreased in cells expressing K562R or K(562,563)R mutants. In TAK1-silenced THP-1 cells, TNF and IL-6 mRNA production in response to LPS was also decreased with reconstitution of K(562,563)R mutant (Supplementary Figure 8A and B). Besides, T(184,187)A mutant that inhibits MAPK signaling also caused inhibition in TNF and IL-6 mRNA production (Fig. 7A,B). To determinate the protein level of cytokines, TAK1-knock down RAW264.7 cells were reconstituted with TAK1 WT, T(184,187)A, K562R, or K(562,563)R mutants. The production of cytokines, such as TNF and IL-6, was detected by ELISA (Fig. 7C,D). In line with the finding that mutation in Lys562 site of TAK1 had a decreased level of mRNA expression of inflammatory cytokines, K562R or K(562,563)R mutant led to the decline of TNF and IL-6 production in response to TLR4 signaling. Furthermore, in line with previous result that Lys562 residue was also critical in TLR2-mediated MAPK activation (Supplementary Figure 6D), the decreased IL-6 mRNA expression could be observed in TAK1-knocked down RAW264.7 cells reconstituted with T(184,187)A or K(562,563)R TAK1 mutants in response to Pam3CSK4 stimulation (Supplementary Figure 8C).

## Discussion

TLR4 signaling is a double-edged sword that protects the host from infection but also causes tissue damage, and therefore requires careful regulation and fine tuning^40^. Through transcriptional regulation of the IKK and MAPK pathways, engagement of TLR4 can induce different levels of inflammatory cytokines and result in different biological outcomes^41^. The basis for this selectivity and specificity remains the biggest challenge in completing understanding of how TLR4 can be engaged in inflammatory signaling. Hitherto, it was also unclear how the two major effectors, IKK and MAPK are differentially regulated. Previously, we demonstrated that the translocation of a multiprotein complex containing MyD88, TRAF6, and TAK1 from the cell membrane to the cytosol compartment where TAK1 is activated is critical for TLR4-mediated MAPKs, but not IKK, activation. In addition to the location of the complex, post-translational modification is also an important factor for signal transduction. Polyubiquitination of TAK1 has been shown to be correlated with activation of TAK1 and is essential for the activation of downstream effectors IKK and the MAPKs upon different immune stimulation.

In this report, we examined the crosstalk between phosphorylation and polyubiquitination of TAK1 and investigated the mechanism through which the distinct activation of MAPKs and IKK is regulated. Although TAK1 inhibitor, 5Z-7-oxozeaenol, that inhibits kinase activity leads to inhibition of both IKK and MAPKs pathways, the results of the reconsititution of TAK1-silenced cells with TAK1 WT or mutant that blocks phosphorylation at Thr184 and Thr187 of the activation loop, suggest that phosphorylated TAK1 is important for MAPKs signaling, but less critical for the IKK pathway in response to LPS. We assume that the basal kinase activity of TAK1 is enough for IKK activation, which needs for further investigation. Moreover, TAK1 phosphorylation status is able to modulate the level of polyubiquitination. By inhibiting TAK1 activation with T(184,187)A mutant or specific inhibitor, 5Z-7-oxozeanenol, TAK1 polyubiquitination is enhanced. Conversely, ubiquitin modification can be counteracted by the TAK1 phospho-mimic mutant, T(184,187)D. These results reveal a feedback loop for phosphorylation and ubiquitination of TAK1, and indicate dynamic regulation of TAK1 polyubiquitiantion and phosphorylation activation. Moreover, Lys562 of TAK1 was identified as a Lys63-linked ubiquitination site and the key residue in the feedback regulation. Mutation in Lys562 of TAK1 leads to inhibition of TAK1 and subsequent MAPK pathway activation.

It has been reported by different groups that TAK1 is modified with Lys63-linked polyubiquitination at specific residues, including Lys34, Lys158 and Lys209, in response to different stimuli. Those reports inferred that regulation of TAK1 occurs in a cell-type specific and ligand specific manner, and indicated the existence of multiple ubiquitination sites. Unlike the Lys63-linked ubiquitination sites^25^,^28^,^30^, the Lys562 residue of TAK1 was identified with mass spectrometry as direct evidence for covalent conjugation between TAK1 and ubiquitin, and was confirmed with *in vivo* ubiquitination assay to be modified with a Lys63-linked polyubiquitin chain. However, the role of TAK1 Lys562 does not conflict with the other ubiquitination sites. Although mutation in the Lys158 residue is unable to reverse the enhancement of polyubiquitination caused by blocking of TAK1 phosphorylation, TAK1 K158R mutant leads to inhibition of TAK1 and the downstream effector, NF-κB pathway^28^. Therefore, in TAK1-mediated signaling, Lys158 of TAK1, which regulates both IKK and MAPK activation, might be more crucial than Lys562. However, Lys562 residue of TAK1 acts as a gatekeeper to trigger TAK1 to activate the MAPKs, and differentially regulates activation of the IKK and MAPK pathways.

**Figure 8:**
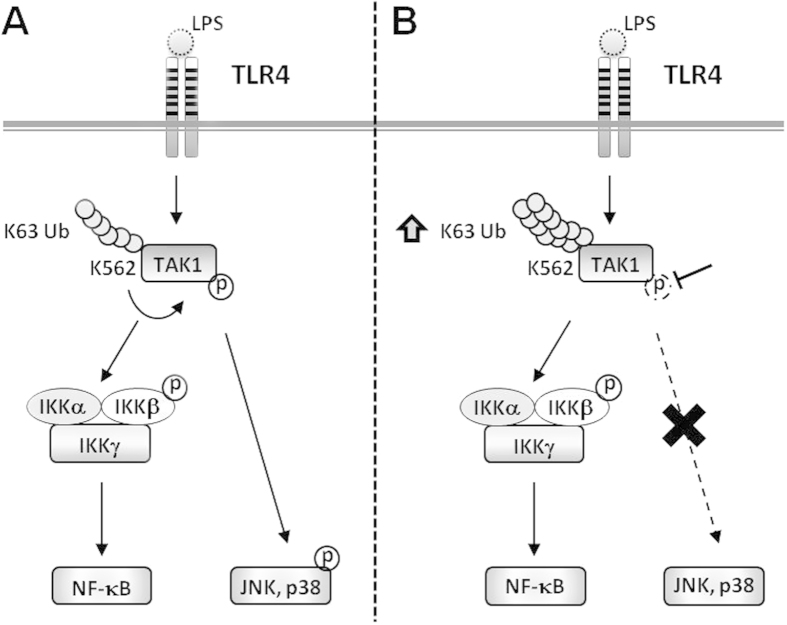
Working model of TAK1 polyubiquitination at Lys562 in TLR4-mediated signaling. ***A***, upon TLR4 engagement, Lys562 residue of TAK1 undergoes Lys63-linked polyubiquitination, and regulates the activation of TAK1 and downstream effectors, JNK and p38. The activated TAK1, in turn, inhibits further modification at Lys562. ***B***, inhibition of the phosphorylation of TAK1 leads to the accumulation of polyubiquitination at Lys562 of TAK1.

In summary, our study demonstrates that the Lys562 residue of TAK1 is a novel Lys63-linked ubiquitinated site that is involved in feedback regulation of TAK1 phosphorylation. As shown in the working model in Figure 8, in response to LPS stimulation, TAK1 undergoes K63-linked ubiquitin modification at Lys562, which is critical for activation of phosphorylation of TAK1 and downstream effectors, JNK and p38. Meanwhile, the activated TAK1, in turn, stops the polyubiquitination at Lys562. Conversely, inhibiting the phosphorylation of TAK1, disregulates the modification at Lys562 and leads to the accumulation of polyubiquitination. Importantly, this report highlights a hitherto unknown feedback regulation loop between polyubiquitination and phosphorylation of TAK1 that dynamically modulates TAK1 activation, and shows how post-translational modification regulates the selection of MAPK activation in TLR4 signaling.

## Methods

### Reagents and materials

The following commercial antibodies were used: HRP-conjugated anti-mouse I gG (NA931), and anti-rabbit IgG (NA941) from GE Healthcare (Pittsburgh, PA), HRP-conjugated anti-rat IgG (AP136P), and anti-ubiquitin Lys63 specific (05–1313) from EMD Millipore (Billerica, MA), anti-TAK1 (sc-7162), anti-IKKα/β.(sc-7607), anti-IκBα.(sc-371), anti-JNK (sc-7345), anti-p38 (sc-728), anti-Tab1 (sc-6052), anti-β-actin (sc-69879), and anti-ubiquitin (sc-8017) from Santa Cruz (Dallas, Texas), anti-p-TAK1 (4531), anti-p-IKKα/β.(2078), anti-p-IκBα.(9246),.anti-p-JNK (9251), and anti-p-p38 (9211) from Cell Signaling (Beverly, MA), anti-HA (11867423001) from Roche (Indianapolis, IN), and anti-Flag (M2, F3165) from Sigma (St. Louis, MO). LPS, TNF, IL-1β,.5Z-7-oxozeaenol, dithiothreitol (DTT), iodoacetamide (IAA), and formic acid were obtained from Sigma. Acetonitrile (ACN) and ammonium bicarbonate were obtained from Aldrich (Milwaukee, WI). Sequencing-grade trypsin was purchased from Promega (Madison, WI).

### Cell culture and treatment

Human HEK293T, human THP-1 and mouse RAW264.7 cells were obtained from American Type Culture Collection (Manassas, VA). The cells were grown in Dulbecco’s modified Eagle’s medium (DMEM) containing 10% fetal bovine serum (FBS), 1 mM sodium pyruvate, and 1% (v/v) penicillin-streptomycin solution, which were from Biological Industries (Beit-Haemek, Israel). For ligand stimulation, cells were washed once with PBS, and incubated with 100 ng/mL LPS, 100 ng/mL Pam3CSK4, 0.1 μg/mL TNF or 0.1 μg/mL IL-1β, respectively, for the indicated times. When TAK1 inhibitor was used, the cells were pre-treated with 100 nM 5Z-7-oxozeaenol for 30 minutes.

### Plasmids

The lentiviral vector, pLU-PL3, for protein expression was a gift from Andrei V. Budanov (Virginia Commonwealth University). pEF-HA-Ub was kindly provided by Ze’ev Ronai (Sanford-Burnham) for *in vivo* ubiquitination assay. pDUO-mMD2/TLR4 was purchased from Invivogen (San Diego, CA), and CMV-Flag-mouse TLR2 was kindly provided by Li-Chung Hsu (National Taiwan University). AP-1 Luc reporter vector from Michael Karin (University of California, San Diego), and pGL4.32[luc2P/NF-κB-RE/Hygro] vector and pRL-TK *Renilla* luciferase control reporter vector purchased from Promega were for luciferase reporter assay. The lentivirus-based specific Map3K7 gene knockdown construct in the pLKO_TRC2 vector, and the packaging plasmids, pCMVΔ8.91 and pMD.G, were from the National RNAi Core Facility (Institute of Molecular Biology/Genomics Research Center, Academia Sinica, Taiwan). The target sequence for 3′UTR region of mouse Map3K7 is as follow: 5′- AGAGAAGACAAACCATTATAA-3′. The target sequence for 3′ UTR region of human MAP3K7 is as follow: 5′- CGGAACCTTTAGGGATAGTTC-3′. Expression vectors encoding specific proteins were constructed by standard recombinant DNA technology. Site-directed mutagenesis was conducted using QuikChange mutagenesis kit from Agilent (Santa Clara, CA). All mutations were confirmed by DNA sequencing.

### Lentivirus production and infection

Lentiviral packaging was as previously described^42^. The 293T cells were transfected with the lentivirus-based constructs along with packaging plasmids, pMD.G and pCMVΔ8.91 by using T-pro NTRII (T-Pro Biotechnology, Taiwan). Virus-containing medium was collected at 48, and 72 hours post-transfection. RAW264.7 or THP-1 cells were infected with lentiviral-containing medium at a multiplicity of infection (MOI) of 10–25 in the presence of 5 mg/ml polybrene (Sigma). After 24 hours, the virus-containing medium was replaced with selection medium containing 5mg/ml puromycin (EMD Millipore). After cell growth was stable, cells were used in subsequent experiments.

### Immunoblotting

Cells were lysed in ice-cold RIPA lysis buffer containing 50 mM Tris-HCl, pH 8.0, 150 mM NaCl, 1% Nonidet P-40, 0.5% deoxycholate, 0.1% SDS, 1 mM phenylmethyl sulfonyl fluoride and 20 μg/ml of aprotinin. After centrifugation, cell extracts were resolved by SDS-PAGE and analyzed by immunoblotting. The membranes were probed with the indicated antibodies. Blots were visualized by chemiluminescence reagent (Thermo, Rockford, IL) exposed on X-ray film (Fujifilm). For densitometric quantification, the Western blot images were analyzed with ImageJ 1.48 (Windows version of NIH Image, http://rsb.info.nih.gov/nih-image/). The relative amount was calculated and the band with the highest intensity was set as 1. The data was presented as mean values from duplicated experiments.

### Immunoprecipitation

Cell lysates were prepared by ice-cold lysis buffer, and immunoprecipitated with the indicated antibodies in immunoprecipitation buffer containing 10 mM Tris-HCl, pH 7.0, 150 mM NaCl, and 0.2% Nonidet P-40 overnight at 4 °C. Whenever protein ubiquitination was analyzed, 20 mM N-ethylmaleimide (NEM, Sigma) was added into the lysis buffer. All samples were incubated with protein G–Sepharose (Roche) for 2 hours at 4 °C. The beads were washed three times with ice-cold lysis buffer or immunoprecipitation buffer, separated by SDS-PAGE and analyzed by immunoblotting with indicated antibodies. Blots were developed with chemiluminescence reagent.

### *In vivo* ubiquitination assays

Flag-tagged protein and HA-tagged ubiquitin were coexpressed in HEK293T cells. 24 hours post transfection, cells were lysed with RIPA lysis buffer containing protease inhibitors and 20 mM NEM (Sigma). The lysate was immunoprecipitated with Flag antibody overnight at 4 °C, and pulled-down with protein G beads. The samples were washed three times with RIPA lysis buffer, gel-separated and analyzed by immunoblotting.

### *In vitro* ubiquitination assays

Flag-tagged TAK1 WT, K562R, or K(562,563)R mutants and Myc-tagged TRAF6 were overexpressed in HEK293T cells, respectively. The tagged proteins were immunoprecipitated with anti-Flag or anti-Myc antibodies. The Flag-tagged proteins were eluted from beads by incubating with 3 × Flag peptide at 4 degrees for 30 mins. The supernatant containing TAK1 proteins was collected and incubated with or without Myc-TRAF6 immunoprecipitated beads in the present of ATP, UBE1, E2 UbcH enzyme set, and either ubiquitin K63 only or ubiquitin K48R mutant (Boston Biochem) for 1 hour. The supernatant and beads were isolated and the reactions were stopped by adding sample buffer and heating at 95 degrees for 2 mins. The supernatant containing TAK1 protein and the beads containing TRAF6 were subjected for immunoblotting analysis.

### Real-time quantitative polymerase chain reaction

Total cellular RNA from 1 × 10^5^ cells was isolated with TRIzol (Invitrogen), and used to synthesize first-strand cDNA with iScript cDNA synthesis kit (Bio-Rad). mRNA amounts were quantified by real-time quantitative polymerase chain reaction (RT-qPCR)^43^ with the StepOnePlus system (Applied Biosystems). Primer sequences are available upon request. All values were normalized to the level of *cyclophilin A* (*PPLA*) mRNA expression.

### The enzyme-linked immunosorbent assay (ELISA)

Cells (3 × 10^5^) were seeded in a 12-well culture plate overnight. After stimulating with the indicated ligands for 24 hours, the cytokine-containing medium was collected, and the protein level was measured with Mouse TNF-alpha DuoSet ELISA Development kit (R&D, Minneapolis, MN) or Mouse IL-6 ELISA set (BD Pharmingen, San Jose, CA).

### Luciferase reporter assays

Cells were seeded into a 96-well culture plate, and co-transfected with either AP-1 reporter or NF-κB reporter pGL4.32, pRL-TK and the indicated expression vectors for 24 hours. The luciferase activity was measured with Dual-Glo luciferase assay system (Promega) following the manufacturer’s instructions.

### Sample preparation for mass analysis

Flag-tagged TAK1 and HA-tagged ubiquitin were co-expressed in HEK293T cells. After 24 hours, cells were collected and underwent *in vivo* ubiquitination assay. Cell lysate with 25 mg total protein was incubated with 250 μl Flag-agarose (Sigma) at 4 ^o^C overnight. Samples were gel-separated, and stained with Coomassie blue. Protein samples containing ubiquinated-TAK1 were recovered from the gel, and subjected to enzyme digestion according to the following procedure. After thermal denaturation at 95 °C for 5 min, 10 μL of protein samples (0.1 mg/mL) were reduced through the addition of DTT to a final concentration of 10 mM and incubated at 50 ^o^C for 30 min. Alkylation was performed by adding IAA to a final concentration of 20 mM. After 30 min of incubation at room temperature in the dark, a second aliquot of DTT was added to quench the reaction. Trypsin solution (20 ng/μL) was added to protein samples (enzyme mixture to sample 1:50, w/w) and the reaction was incubated at 25 ^o^C for 12 h. The enzymatic digestions were quenched through the addition of a 10 μL formic acid (10%). Digested peptides were dried by vacuum concentrator (Savant SPD131DDA, Thermo Fisher) prior to mass analysis.

### Liquid chromatography-mass spectrometry

Mass spectral data were acquired using a Thermo LTQ-FT mass spectrometer (Thermo Fisher, Santa Clara, CA) equipped with a nanoelectrospray ion source (New Objective, Woburn, MA), an Agilent 1100 Series binary high-performance liquid chromatography pump (Agilent Technologies, Palo Alto, CA), and a Famos autosampler (LC Packings, San Francisco, CA). The vacuum-dried digested samples were reconstituted with mobile phase A buffer and 5 μL of samples were injected on to a self-packed pre-column (150 μm I.D. × 30 mm, 5 μm, 100 Å) at a flow rate of 10 μL/min. Chromatographic separation was performed on a self-packed reversed phase C18 nano-column (75 μm I.D. × 200 mm, 3 μm, 100 Å) using 0.1% formic acid in water as mobile phase A and 0.1% formic acid in 80% acetonitrile as mobile phase B operated at the flow rate of 300 nL/min. Separation was attained using a solvent gradient ramping from 10% to 40% of mobile phase B in 45 min. The mass scanning range was set from *m/z* 350 to 2,000 for the survey full-scan MS mode and data dependent MS/MS acquisition. The ten most abundant ions detected in a full-scan were subjected to a MS/MS experiment performed in a LTQ mass spectrometer. Ion accumulation (automatic gain control target number) and maximal ion accumulation time for a full-scan and MS/MS were set at 1 × 10^6^ ions, 1000 ms, and 5 × 10^4^ ions, 200 ms. Ions were fragmented by use of collision induced dissociation (CID) with the normalized collision energy set to 35%, activation Q at 0.3 and activation time at 30 ms. For data analysis, all MS/MS spectra were converted to mzXML and mgf format from the experimental RAW file using MM File Conversion Tools (http://www.massmatrix.net)^44^, and then analyzed by MassMatrix for MS/MS ion search^45^. The search parameters in MassMatrix including the error tolerance of precursor ions and the MS/MS fragment ions in spectra were 10 ppm and 0.6 Da and the enzyme was assigned as trypsin with three missed cleavages allowed. The variable post-translational modifications in the search parameters were assigned to include the oxidation of methionine, carbamidomethylation of cysteine, phosphorylation of serine/threonine/tyrosine, and acetylation of lysine.

### Statistical analysis

Data are expressed as the mean ± SD. All statistical analyses were conducted using Prism (version 6.01). The significance differences between groups were determined by Dunnett t-test with unequal variances between groups. (*, significant difference)

## Additional Information

**How to cite this article**: Chen, I.-T. *et al.* Polyubiquitination of Transforming Growth Factor β-activated Kinase 1 (TAK1) at Lysine 562 Residue Regulates TLR4-mediated JNK and p38 MAPK Activation. *Sci. Rep.* **5**, 12300; doi: 10.1038/srep12300 (2015).

## Supplementary Material

Supplementary Information
