# Supplementary material for: Polyubiquitination of Transforming Growth Factor β-activated Kinase 1 (TAK1) at Lysine 562 Residue Regulates TLR4-mediated JNK and p38 MAPK Activation
Source: Sci Rep. 2015 Jul 20;5:12300. doi: 10.1038/srep12300 (PMC4507259; doi:10.1038/srep12300)

SUPPLEMENTARY INFORMATION

Polyubiquitination of Transforming Growth Factor β-activated Kinase 1 (TAK1) at Lysine 562 Residue Regulates TLR4-mediated JNK and p38 MAPK Activation

I-Ting Chen[1], Pang-Hung Hsu[1,2], Wan-Ching Hsu[1], Nien-Jung Chen[3,4], and Ping-Hui Tseng[1,4*]

Supplementary Figure legends

Supplementary Figure 1. TAK1 is required for IKK and MAPK signaling. *A*, TAK1 is required for IKK and MAPK activation. TAK1 in human THP-1 cells was knocked down with lentiviral-based shRNA. After treatment with LPS (100 ng/mL) for the indicated times, cells were lysed and subjected to immunoblot analysis. *B* and *C*, TAK1 is required for TLR4-mediated TNF and IL-6 production. Control and TAK1-silenced THP-1 cells were stimulated with LPS for indicated times, and total RNA was extracted. After reverse transcription, TNF and IL-6 mRNA were analyzed with Q-PCR. Results of Q-PCR are averages ± SD of three separate experiments (*, *significant difference*).

Supplementary Figure 2. IKK and MAPK activation is blocked by TAK1 inhibition. *A*, after treatment with TAK1 inhibitor 5Z-7-oxozeanenol (100 nM) for 30 min, RAW264.7 cells were stimulated with LPS for indicated times. The cells were lysed and kinase activation was analyzed by immunoblotting. *B* and *C*, TAK1 inhibition decreased TLR4-mediated TNF and IL-6 mRNA production in RAW264.7 cells. RAW264.7 cells were pretreated with TAK1 inhibitor 5Z-7-oxozeanenol (100 nM) for 30 min, and stimulated with LPS for indicated times. Total RNA was extracted, and TNF and IL-6 mRNA were analyzed with Q-PCR. *D* and *E*, TAK1 inhibition decreased TLR4-mediated TNF and IL-6 mRNA production in THP-1 cells. THP-1 cells were pretreated with TAK1 inhibitor and stimulated with LPS for indicated times. Total RNA was extracted, and TNF and IL-6 mRNA were analyzed with Q-PCR. Results of Q-PCR are averages ± SD of three separate experiments (*, *significant difference*).

Supplementary Figure 3. Lys63-linked polyubiquitination of TAK1 is enhanced by blocking TAK1 phosphorylation. In response to LPS, TAK1 polyubiquitnation is enhanced by inhibiting TAK1 activation. *A*, RAW264.7 cells and *B*, THP-1 cells were pretreated with TAK1 inhibitor 5Z-7-oxozeanenol and incubated with LPS for the indicated times. After immunoprecipitation, polyubiquitination of TAK1 was detected with anti-Lys63-linked Ub antibodies.

Supplementary Figure 4. Phosphorylation of TAK1 at Thr184 and Thr187 residues is critical for the activation of MAPKs. Flag-tagged TAK1 wild-type (WT), T(184,187)A or T(184,187)D mutant, Renilla-Luc plasmid and AP-1 reporter were co-expressed in HEK293T cells for 24 h. Relative luciferase activity was measured and normalized with the Renilla activity. Results are averages ± SD of three separate experiments (*, *significant difference*).

Supplementary Figure 5. Lys562 residue of TAK1 is a Lys63-linked ubiquitination site. *A*, in response to LPS, Lys562 or Lys563 residue of TAK1 undergoes Lys63-linked polyubiquitination. TAK1-silenced THP-1 cells were reconstituted with Flag-tagged TAK1 wild-type (WT) or K(562,563)R mutant. The cells were incubated with LPS for the indicated times, lysed, immunoprecipitated, and detected polyubiquitination with anti-Lys63-linked Ub antibodies. *B* and *C*, Lys562 or Lys563 residue of TAK1 is critical for Lys63-linked polyubiquitination with in vitro ubiquitination assay. Flag-tagged TAK1 WT, K562R, or K(562,563)R mutants was incubated with Myc-TRAF6 immunoprecipitated beads in the present of ATP, UBE1, E2 UbcH enzymes, and either

ubiquitin K63 only or ubiquitin K48R mutant for 1 hour. The supernatant containing TAK1 protein and the beads containing TRAF6 were isolated and subjected for immunoblotting analysis. *D*, in response to Pam3CSK4, Lys562 or Lys563 residue of TAK1 undergoes Lys63-linked polyubiquitination. TAK1-silenced RAW264.7 cells were reconstituted with Flag-tagged TAK1 wild-type (WT) or K(562,563)R mutant. The cells were incubated with Pam3CSK4 for the indicated times, lysed, immunoprecipitated, and detected polyubiquitination with anti-Lys63-linked Ub antibodies.

Supplementary Figure 6. Polyubiquitination of TAK1 at Lys562 is required for the activation of TAK1 and MAPK, but not IKK. *A*, mutation in Lys562 residue of TAK1 inhibits the activation of TAK1 and MAPK, but not IKK, in response to LPS. TAK1-silenced THP-1 cells were reconstituted with Flag-tagged TAK1 wild-type (WT) or K(562,563)R mutant. The cells were incubated with LPS for the indicated times, and phosphorylated proteins were analyzed with immunoblotting. *B* and *C*, mutation in Lys562 residue of TAK1 inhibits the activation of TAK1 and MAPK, but not IKK, with stimulation of IL-1beta and TNF. RAW264.7 cells were overexpressed with Flag-tagged TAK1 wild-type (WT) or K(562,563)R mutant. The cells were stimulated with *B*, IL-1beta and *C*, TNF for the indicated times, lysed and phosphorylated proteins were analyzed with immunoblotting. *D*, mutation in Lys562 residue of TAK1 inhibits the activation of MAPK in response to Pam3CSK4. Flag-tagged TAK1 wild-type (WT), K562R or K(562,563)R mutant, Renilla-Luc plasmid and AP-1 reporter were co-expressed with or without TLR2 in HEK293T cells for 24 h. Relative luciferase activity

was measured and normalized with the Renilla activity. Results are averages ± SD of three separate experiments (*, *significant difference*).

Supplementary Figure 7. Mutation in Thr184 and 187 residues of TAK1 does not disrupt the interaction of TAK1 with TRAF6. Flag-tagged TAK1 wild-type (WT), or T(184,187)A mutant were co-expressed in HEK293T cells with myc-tagged TRAF6. The cells were lysed and immunoprecipitated with anti-Flag antibody. After gel separation, TAK1-containing complex was detected with the indicated antibodies.

Supplementary Figure 8. *A* and *B*, TAK1 is required for TLR4-mediated induction of TNF and IL-6 mRNA. TAK1-silenced THP-1 cells were reconstituted with Flag-tagged TAK1 wild-type (WT), or K(562,563)R mutant. After treatment with LPS for indicated times, the cells were collected and the total RNA was extracted, reverse transcribed, and analyzed for TNF and IL-6 mRNA with Q-PCR. *C*, TAK1 is required for TLR2-mediated induction of IL-6 mRNA. TAK1-silenced RAW264.7 cells were reconstituted with Flag-tagged TAK1 wild-type (WT), T(184,187)A or K(562,563)R mutants. After treatment with Pam3CSK4 for indicated times, the cells were collected and the total RNA was extracted, reverse transcribed, and analyzed for IL-6 mRNA with Q-PCR. Results are averages ± SD of three separate experiments (*, *significant difference*).

Supplementary Figure 1

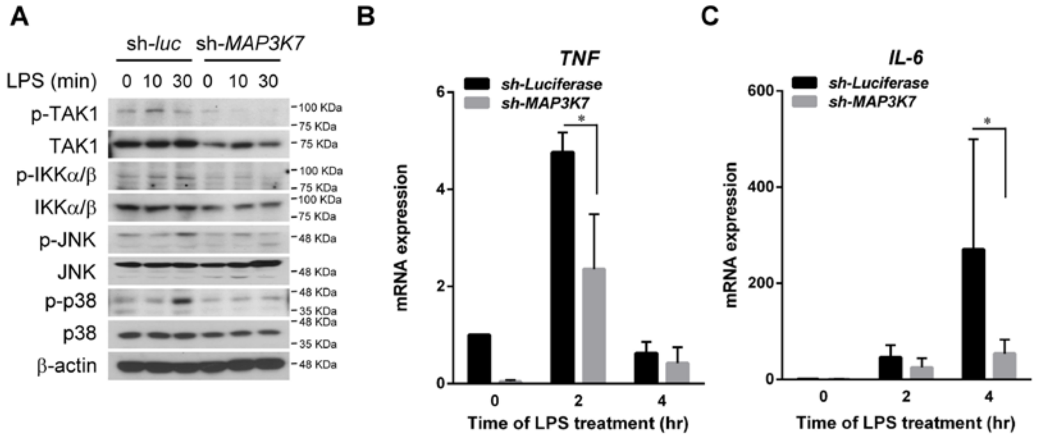

Supplementary Figure 2

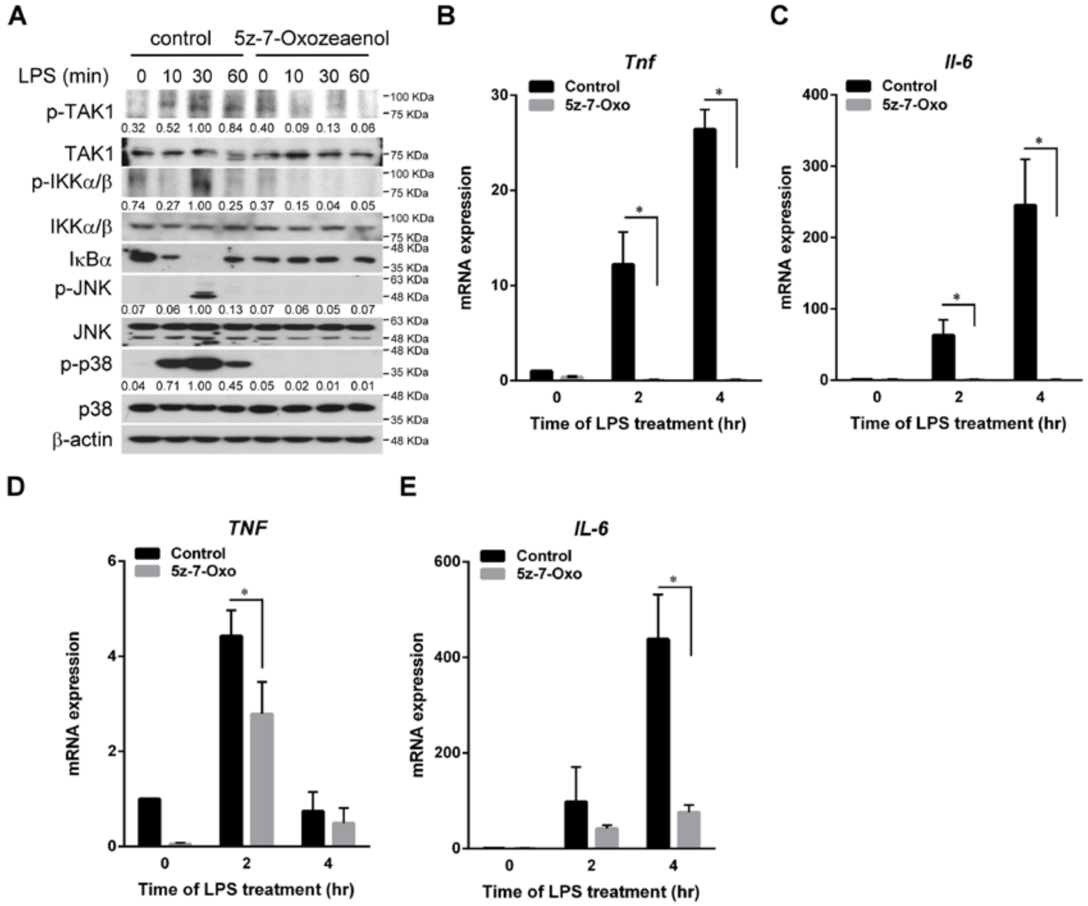

Supplementary Figure 3

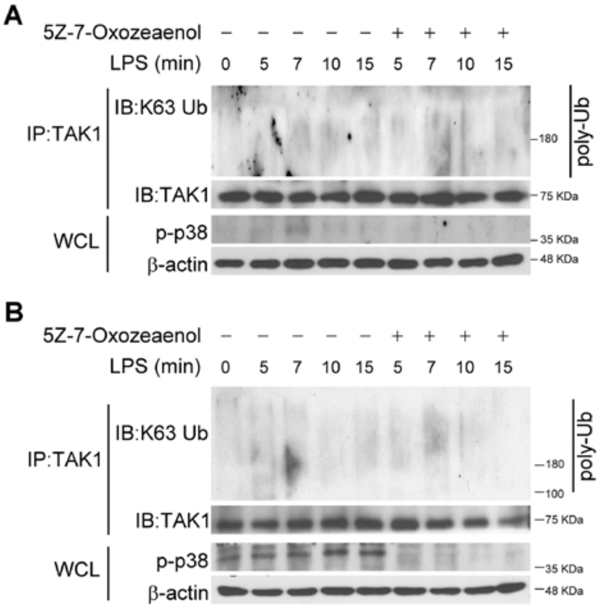

Supplementary Figure 4

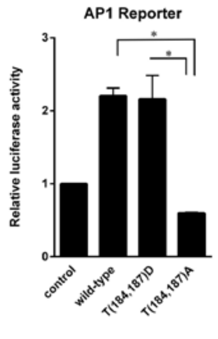

Supplementary Figure 5

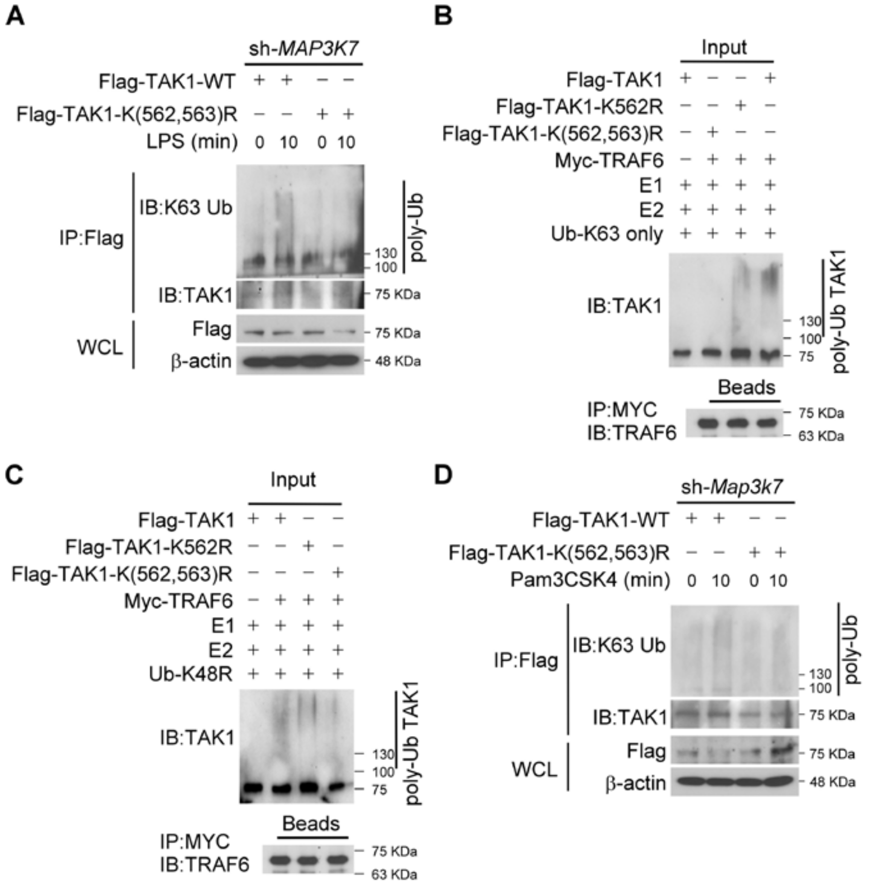

Supplementary Figure 6

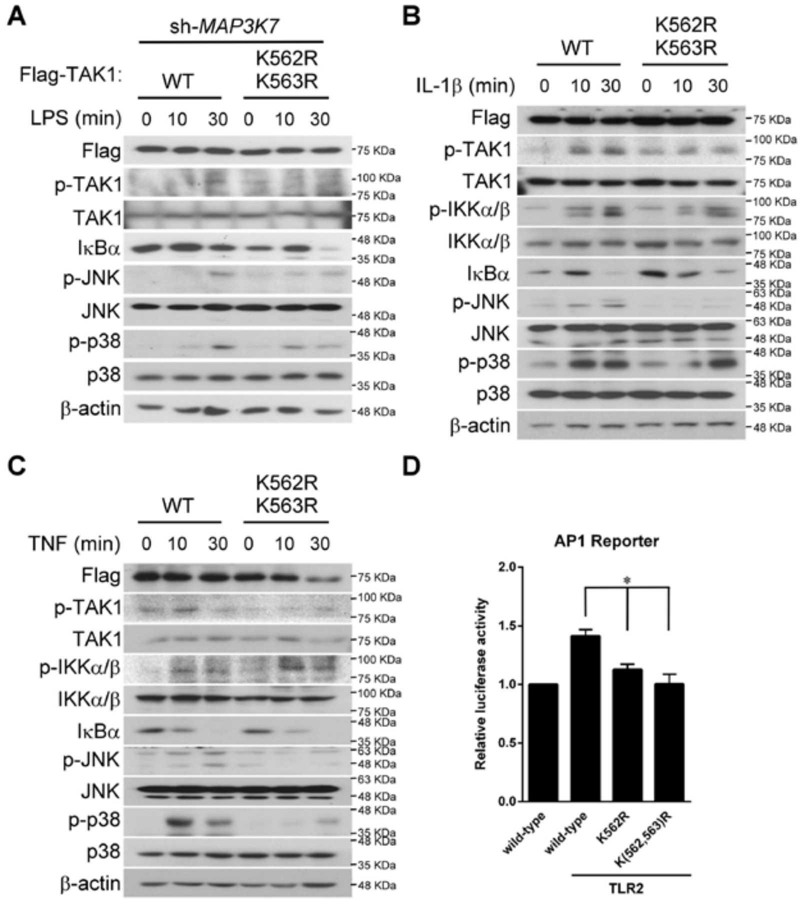

Supplementary Figure 7

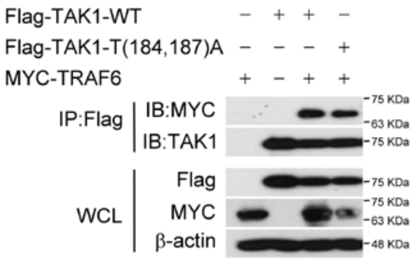

Supplementary Figure 8

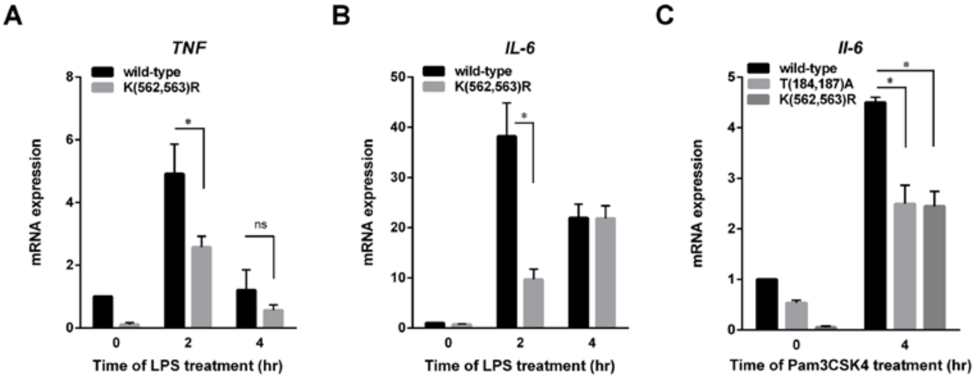

Original blots before cropping for main figures

Fig 1A

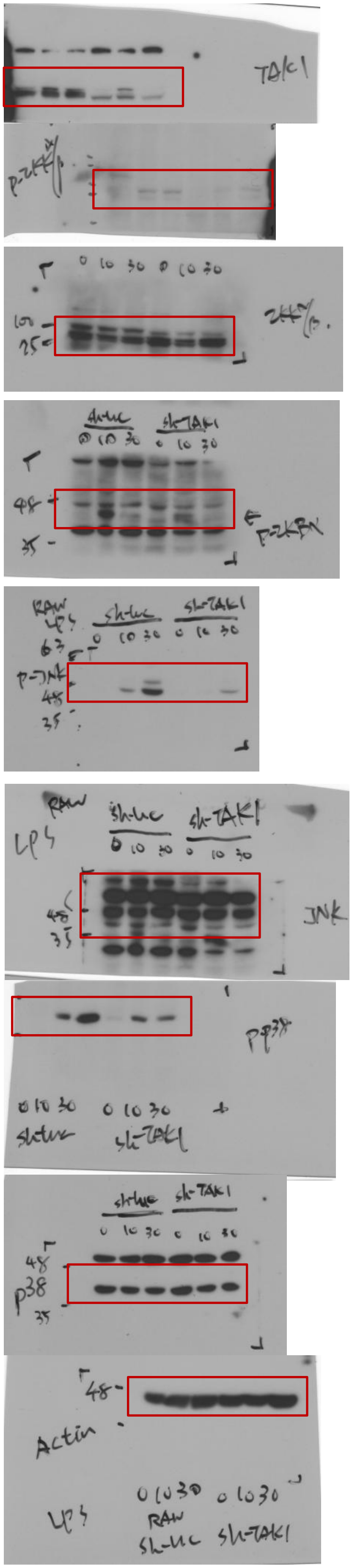

Fig 1E

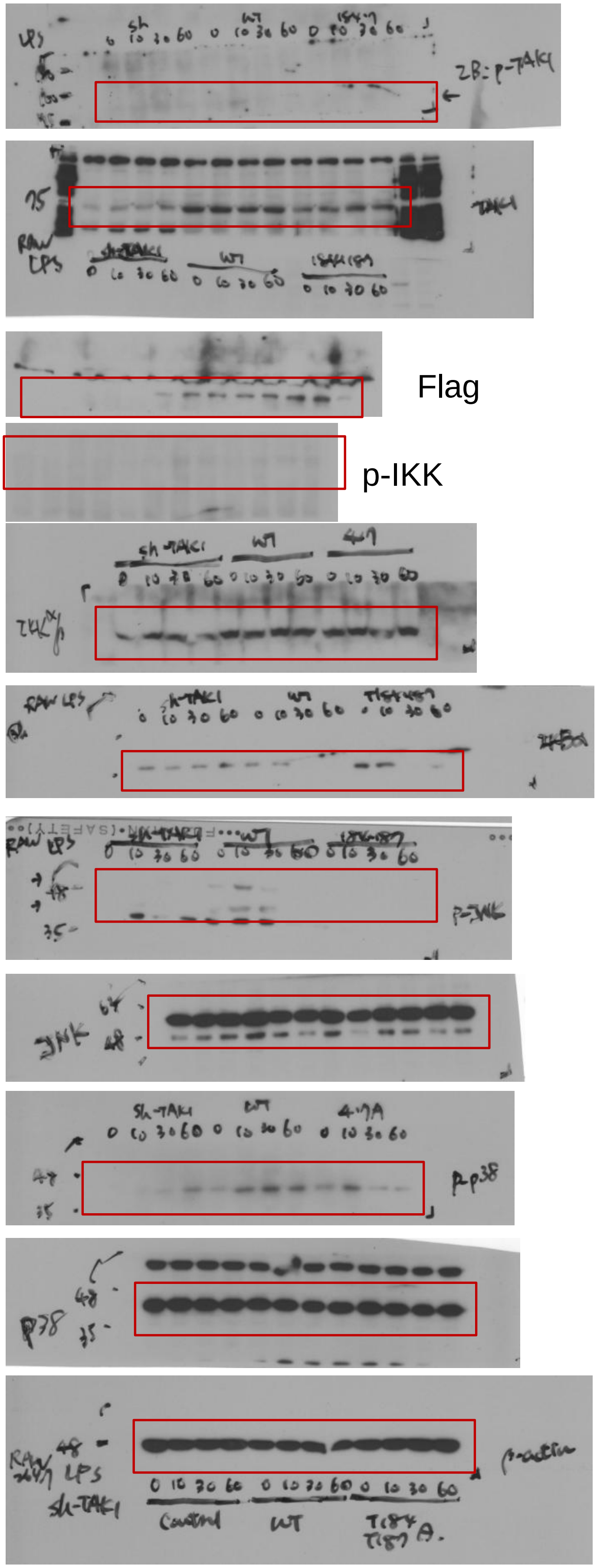

Fig 2A

WT-Ub

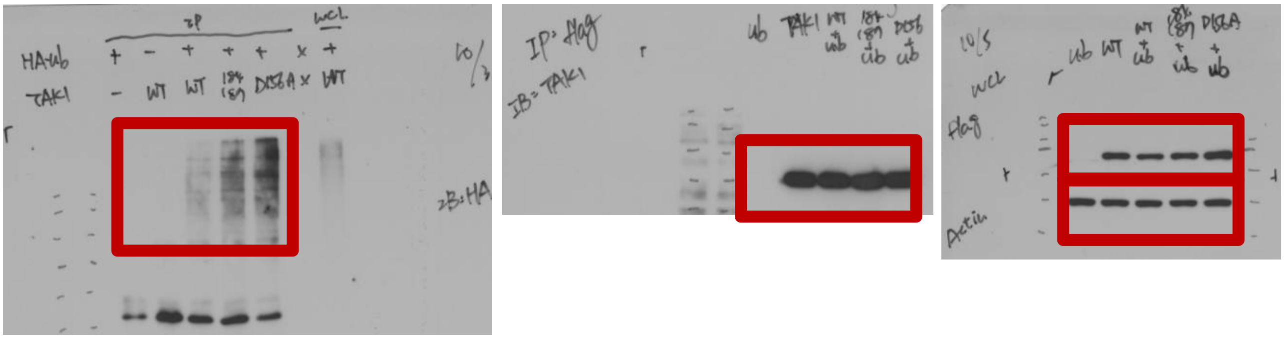

K48R-Ub

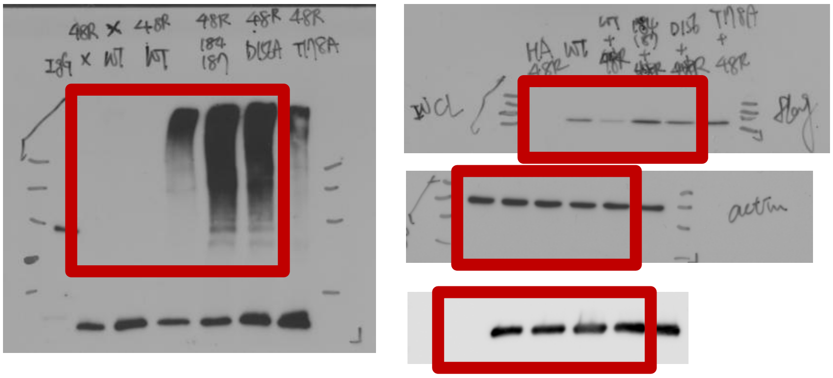

K63R-Ub

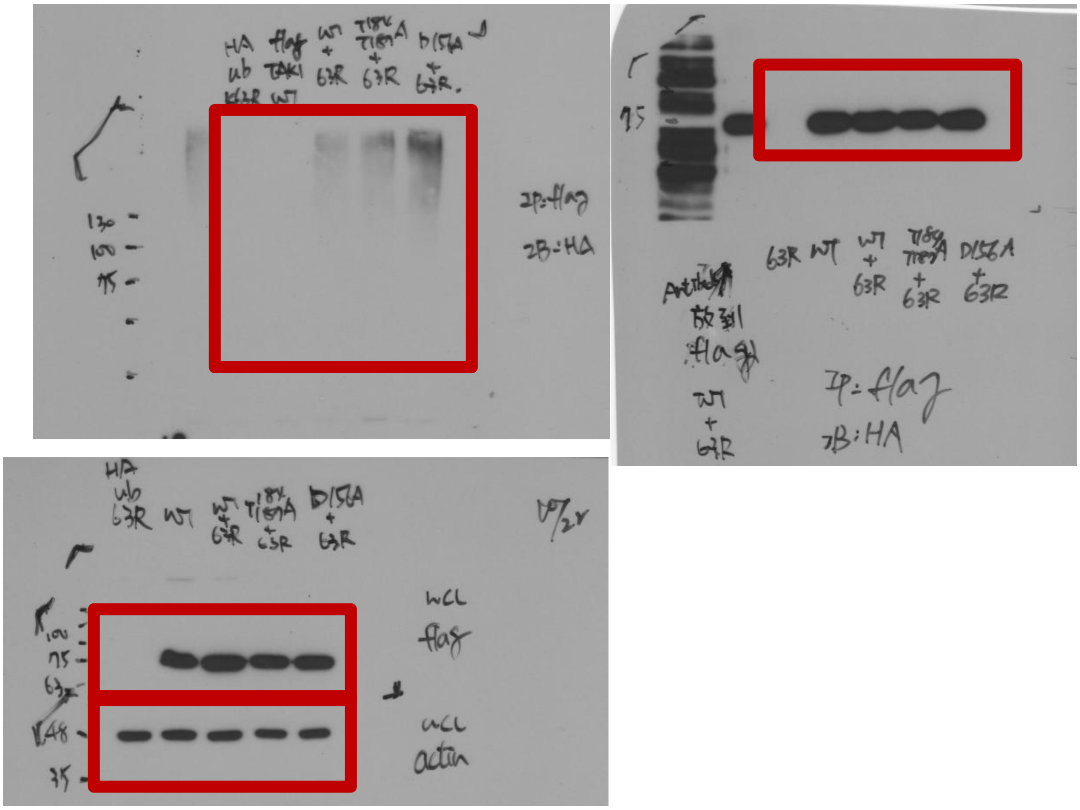

Fig 2B

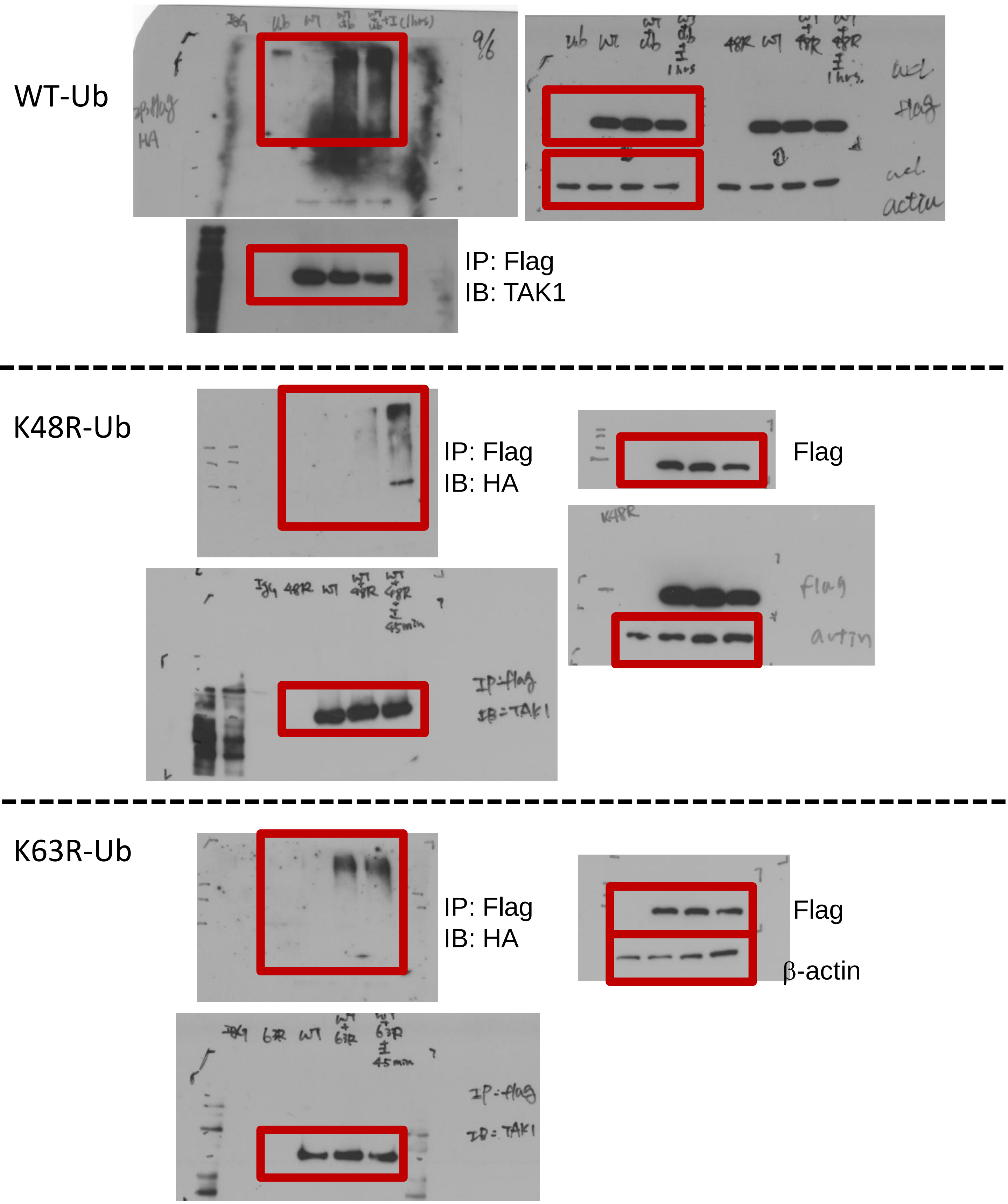
WT-Ub
IP: Flag
IB: TAK1
K48R-Ub
IP: Flag
IB: HA
Flag
K63R-Ub
IP: Flag
IB: HA
Flag
β-actin

Fig 2C

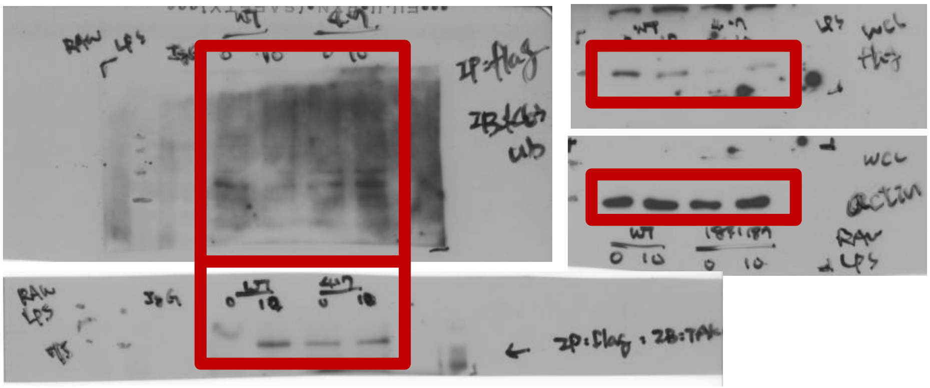

Fig 2D

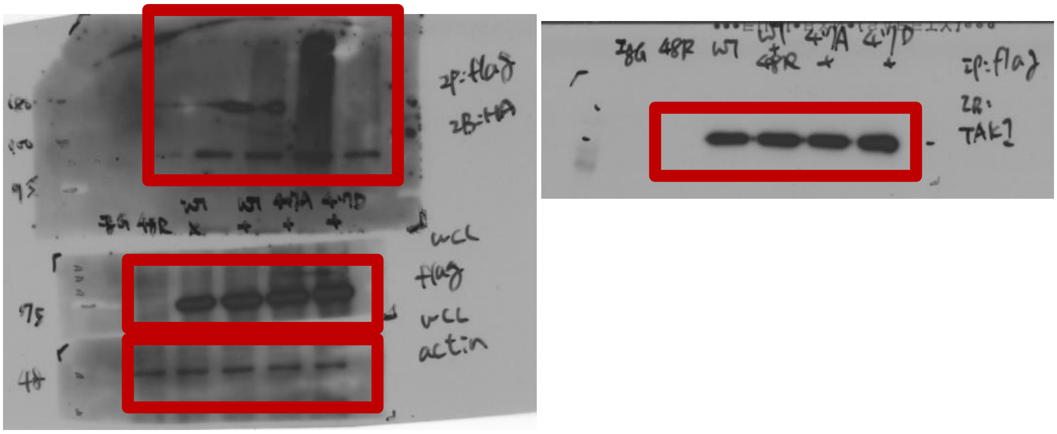

Fig 2E

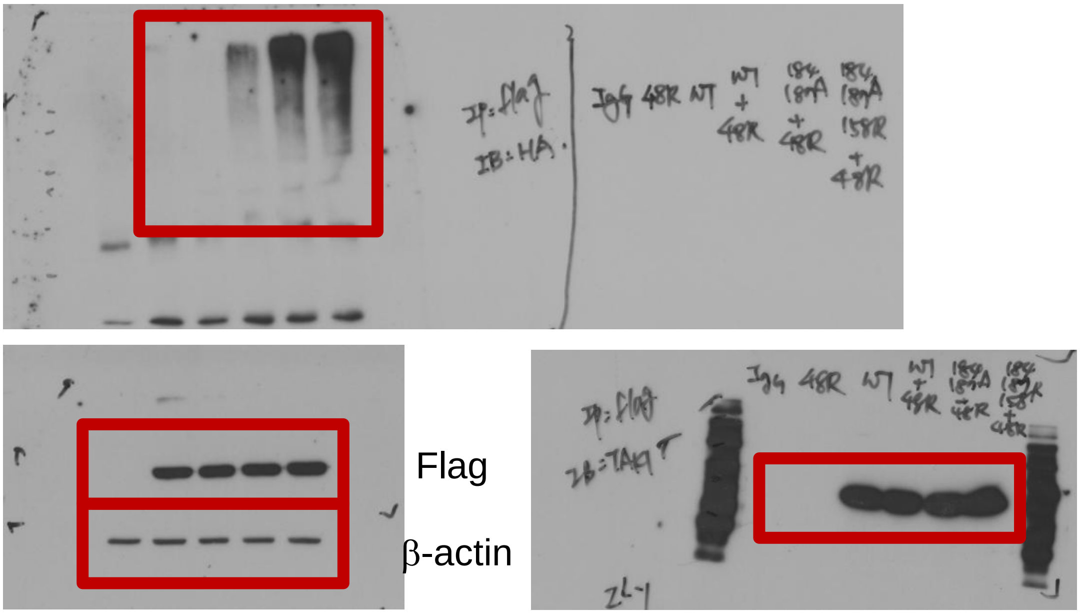

Fig 4A

WT-Ub

K48R-Ub

K63R-Ub

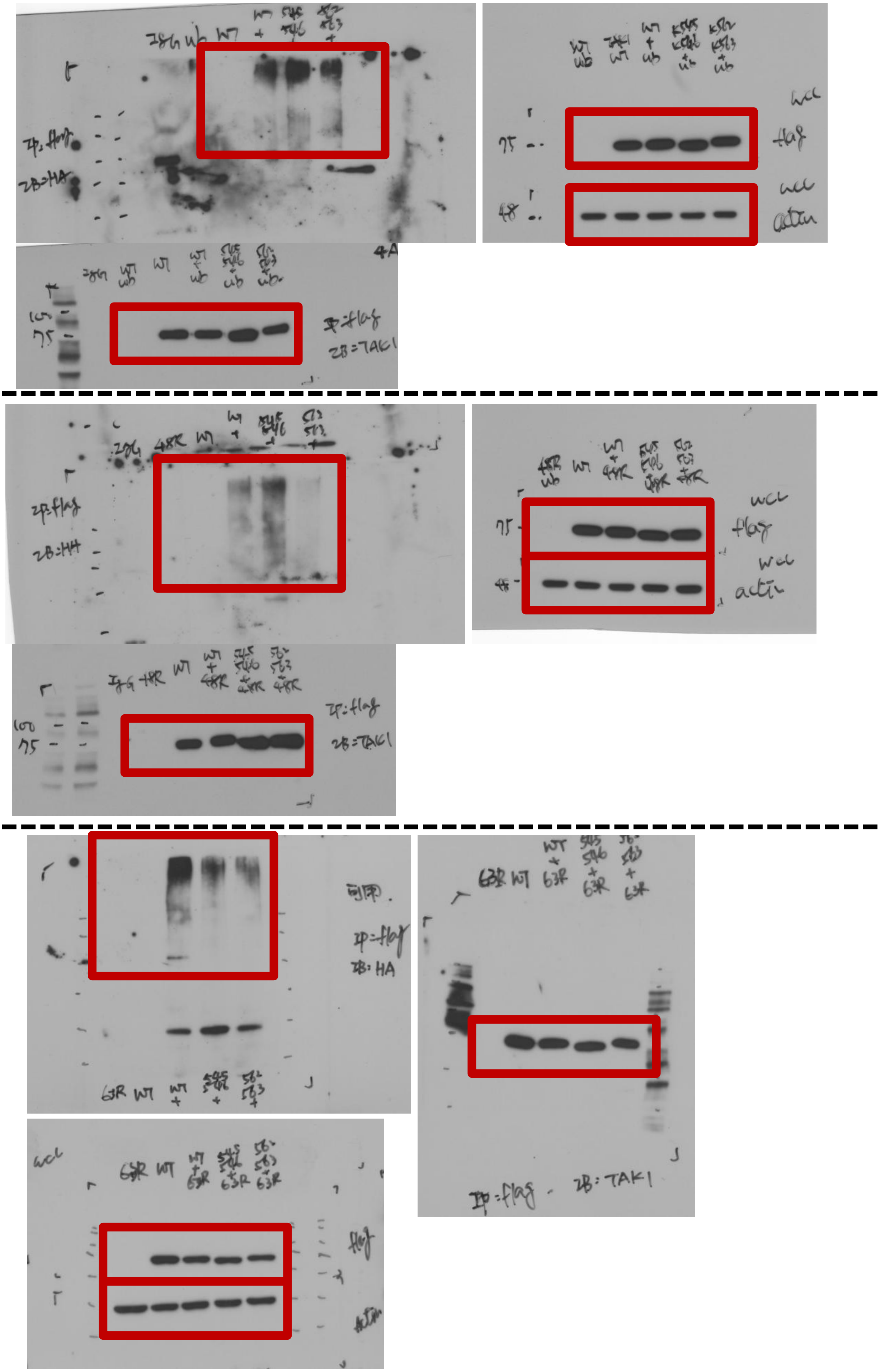

Fig 4B

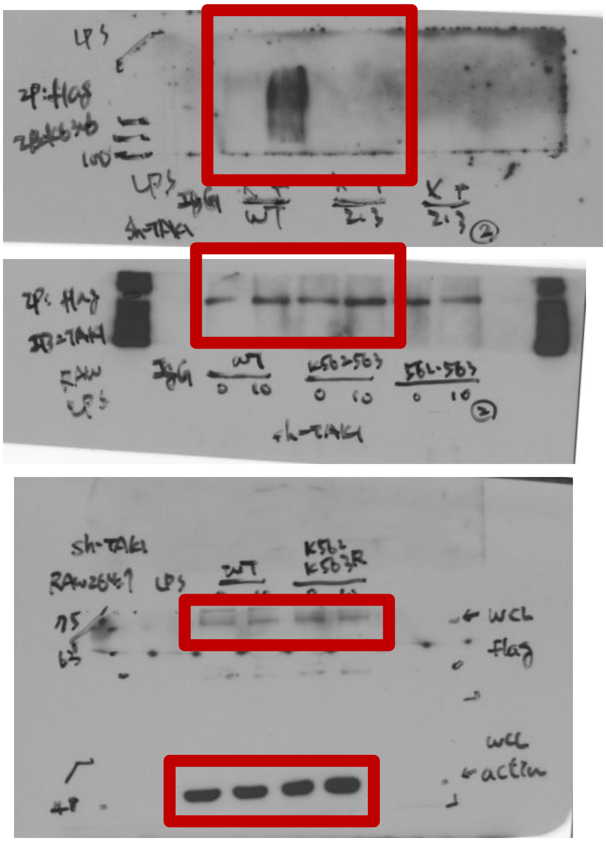

Fig 4C

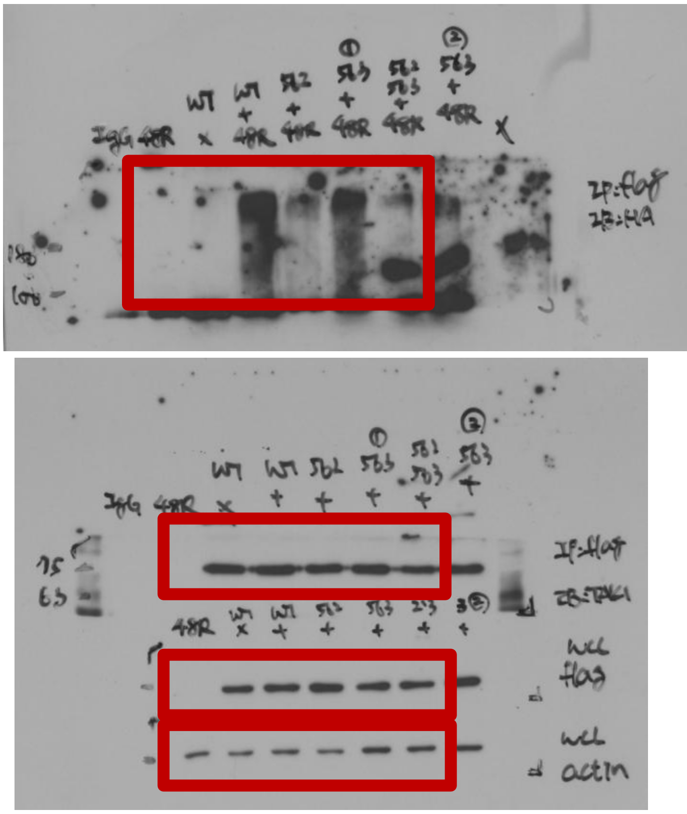

Fig 4D

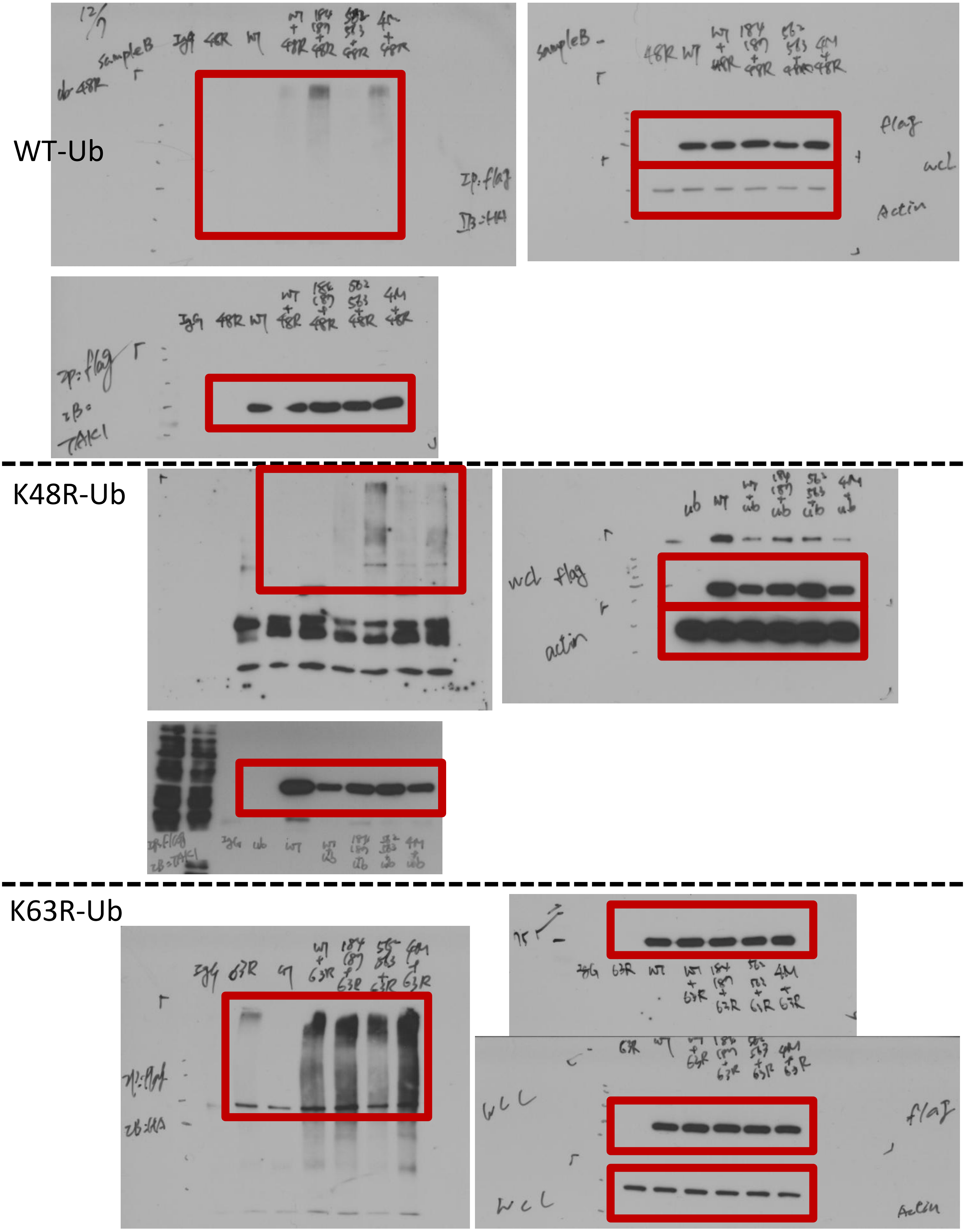

Fig 4E

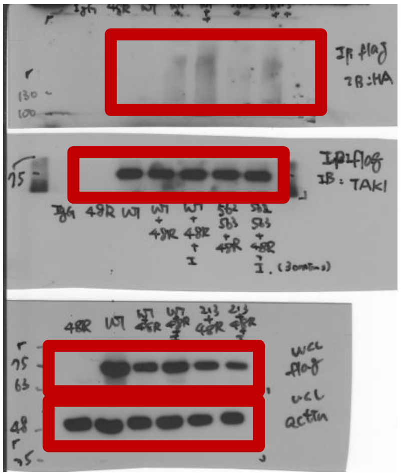

Fig 4F

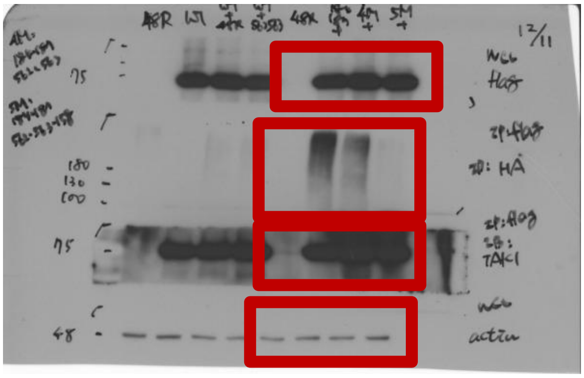

Fig 5A

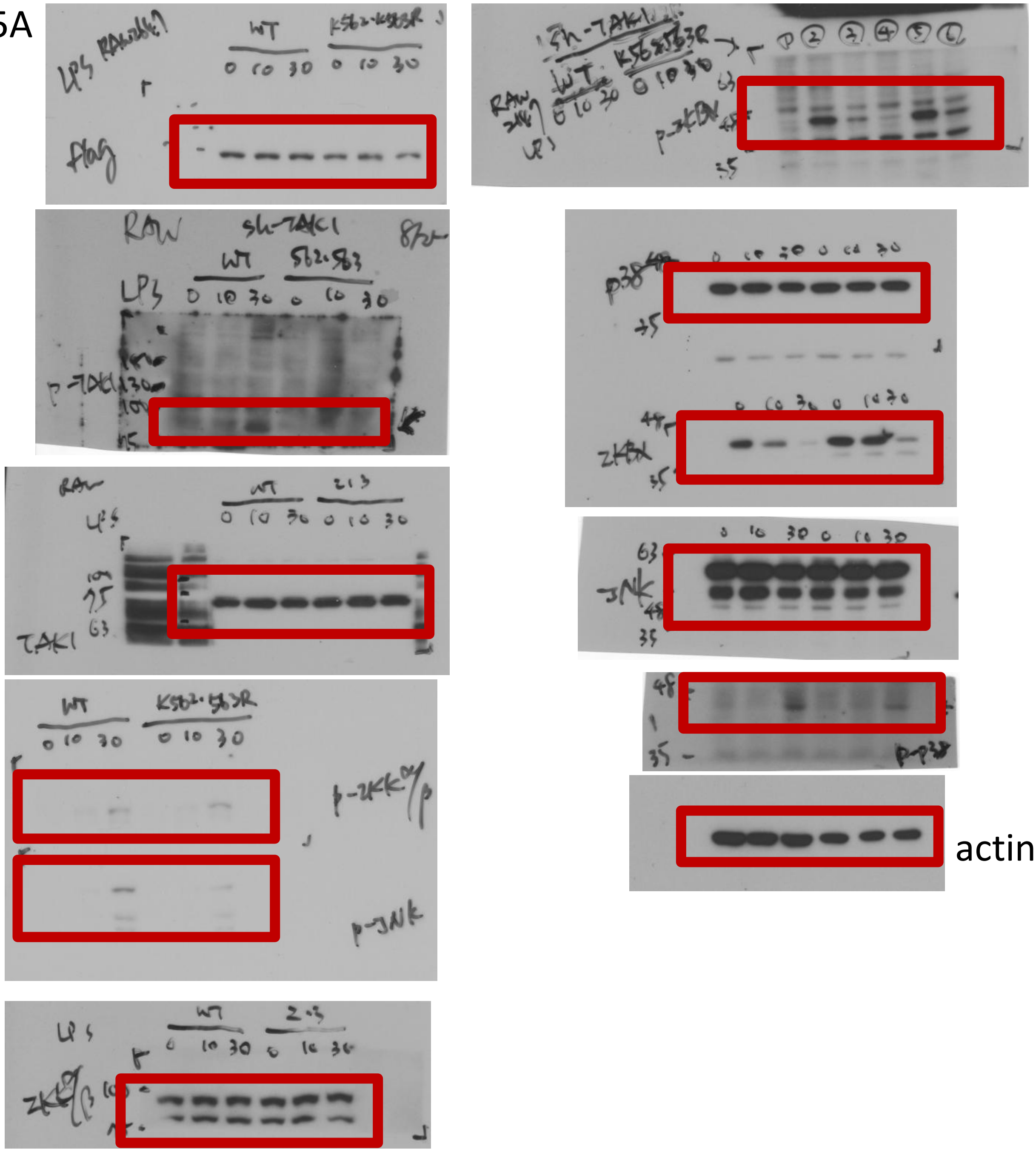

actin

Fig 6A

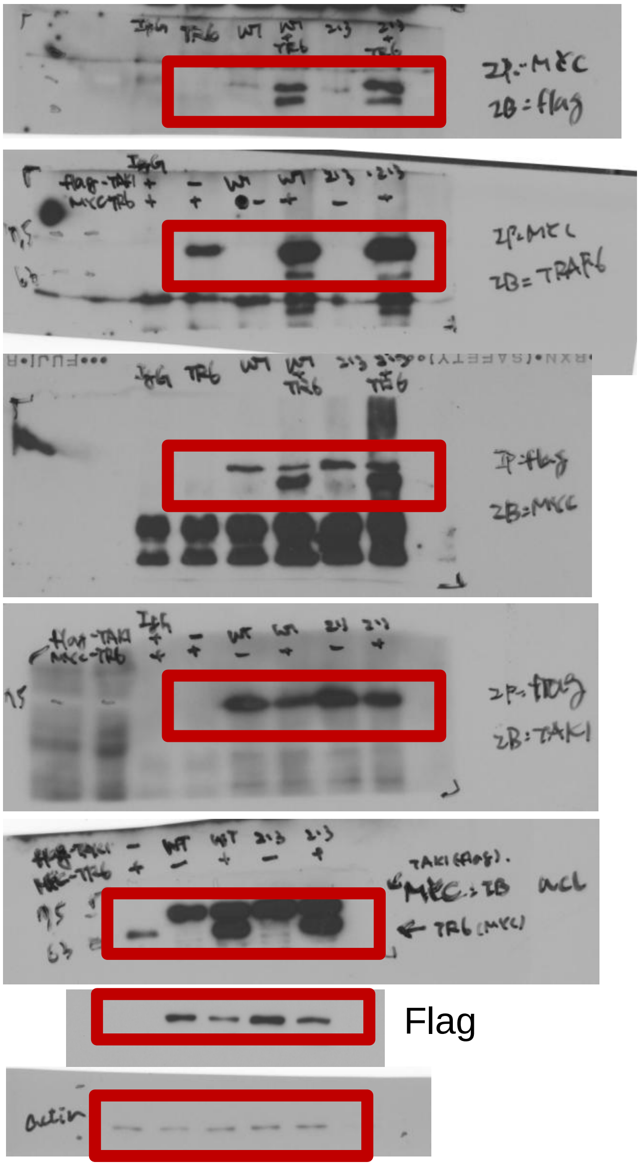

Fig 6B

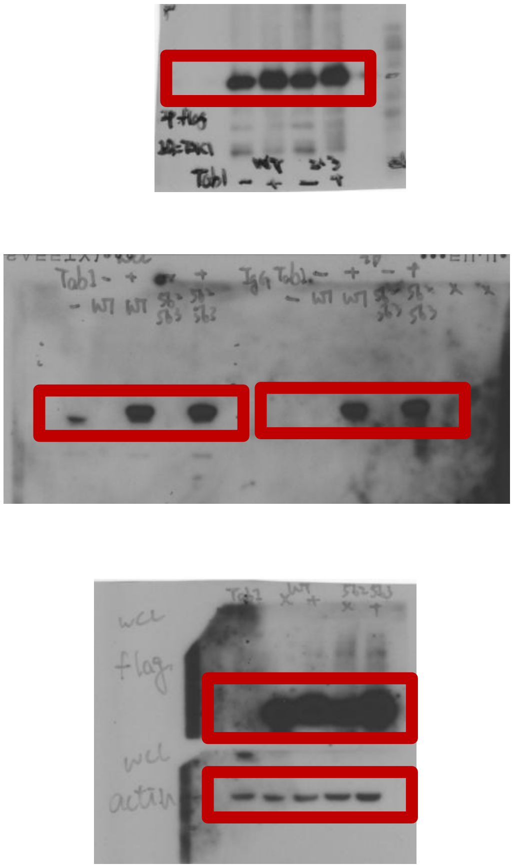

Fig 6C

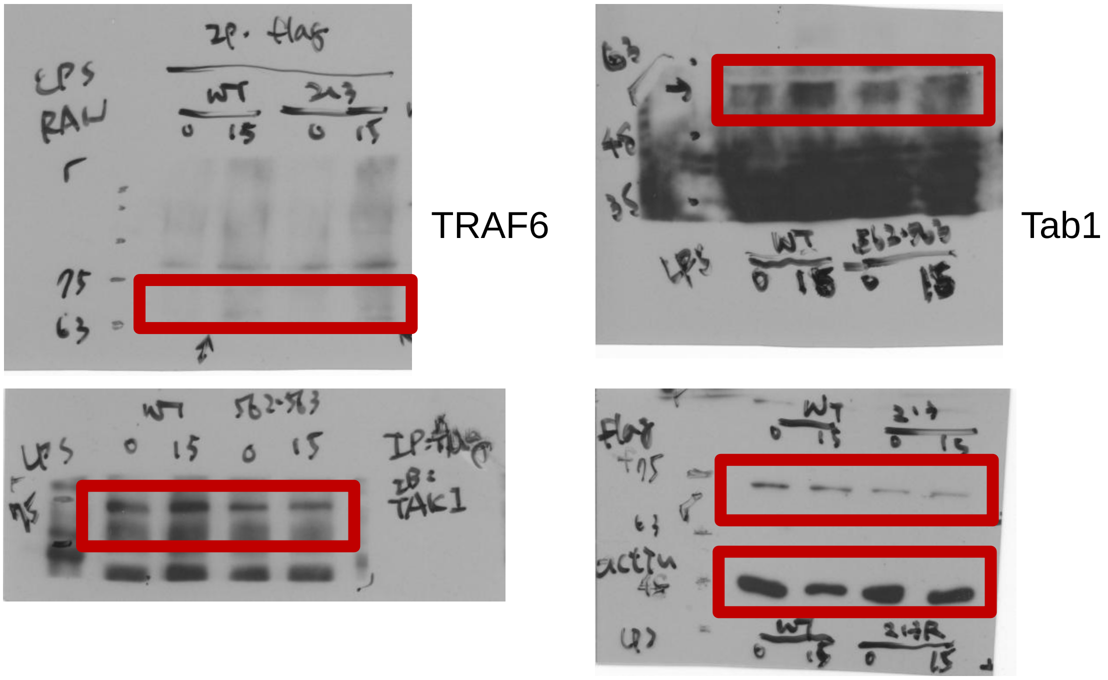

Suppl. Fig1A

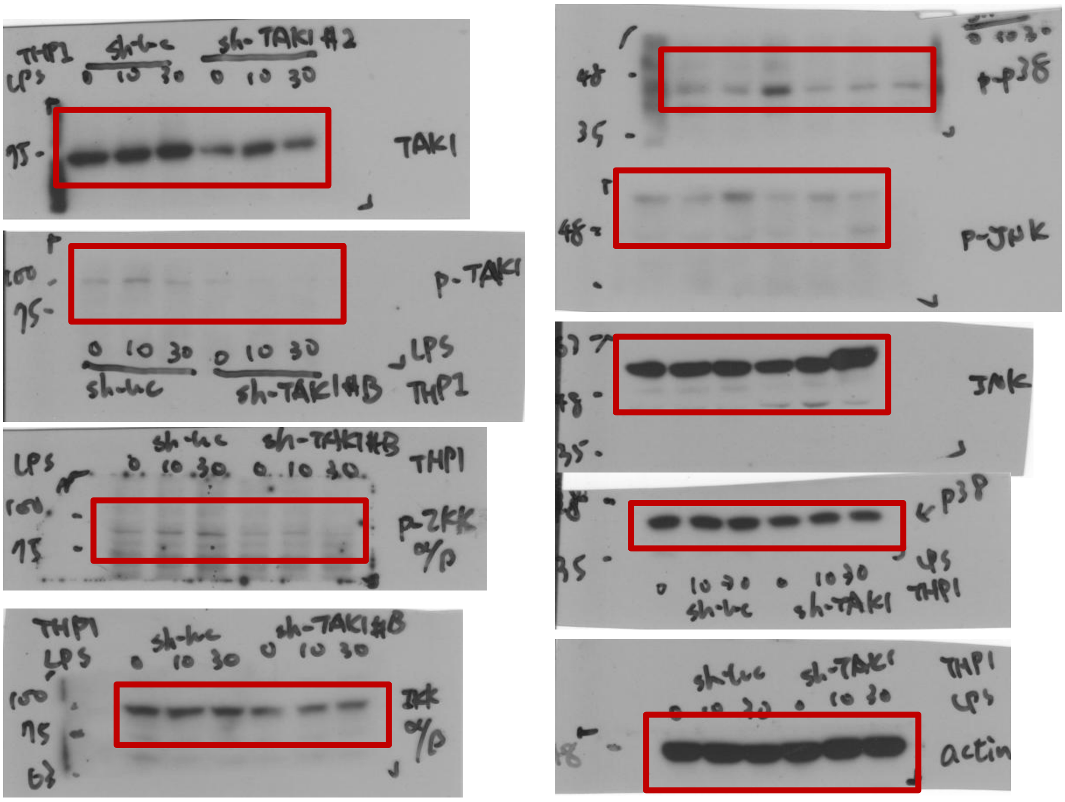

Suppl. Fig2A

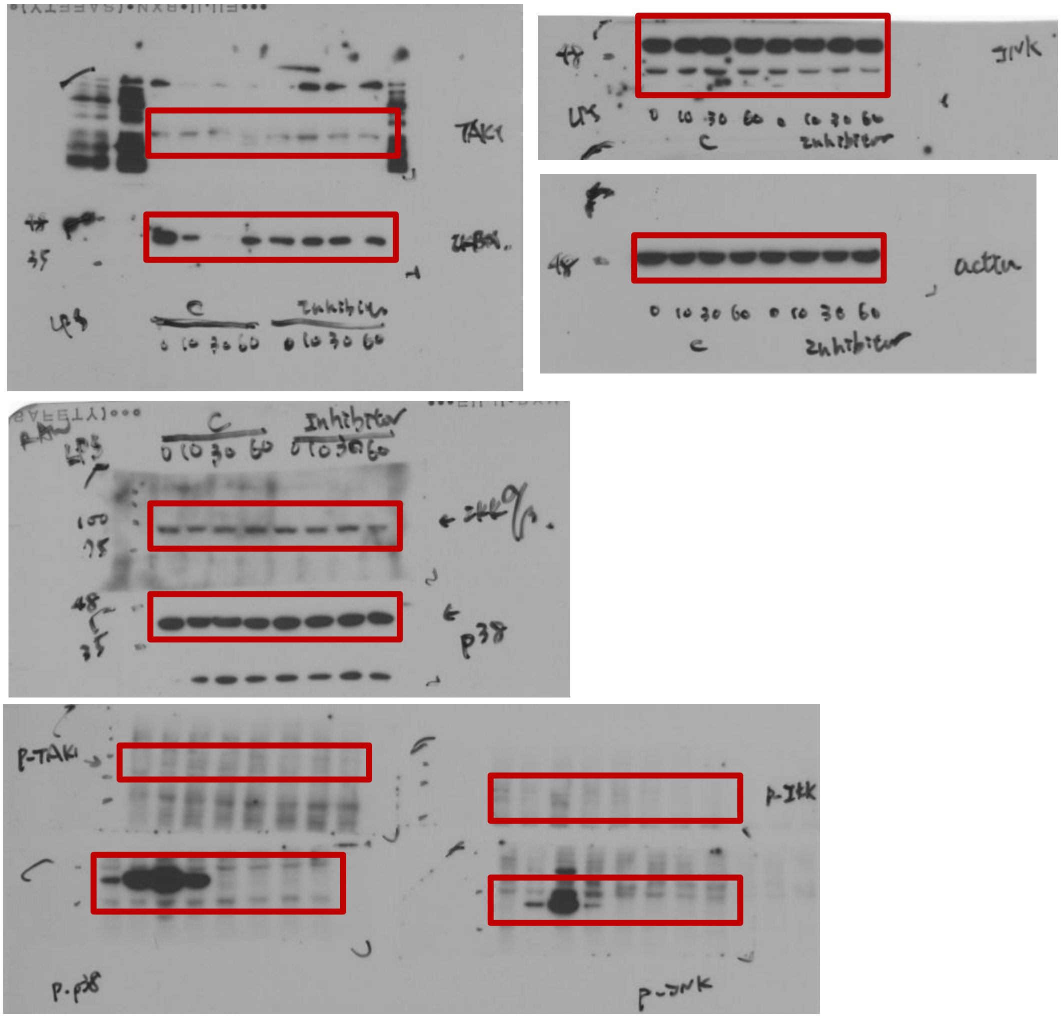

Suppl. Fig3A

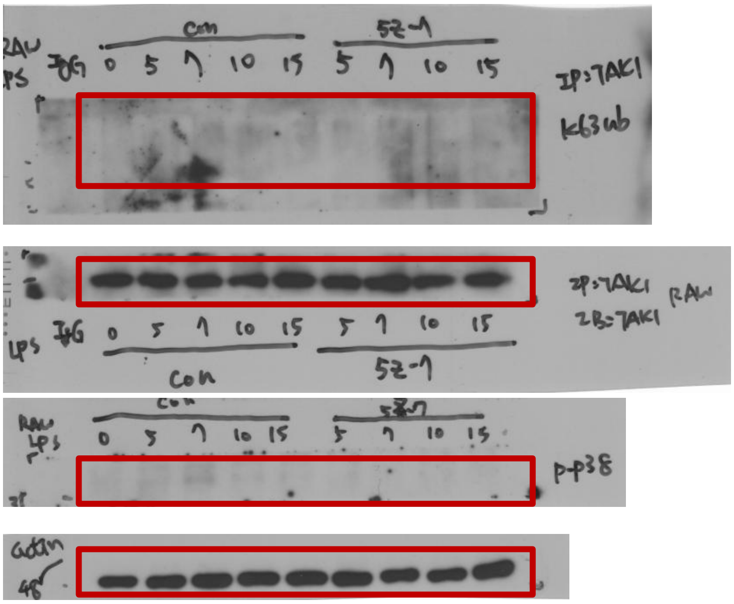

Suppl. Fig3B

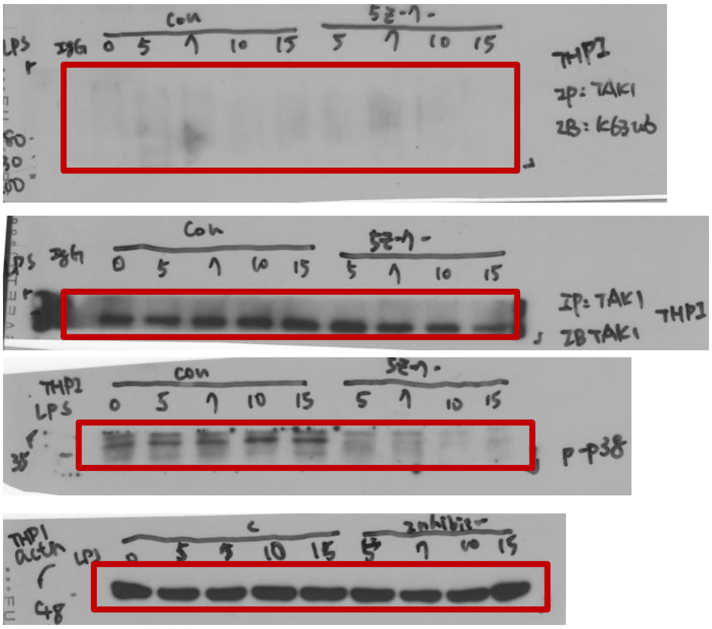

Suppl. Fig5A

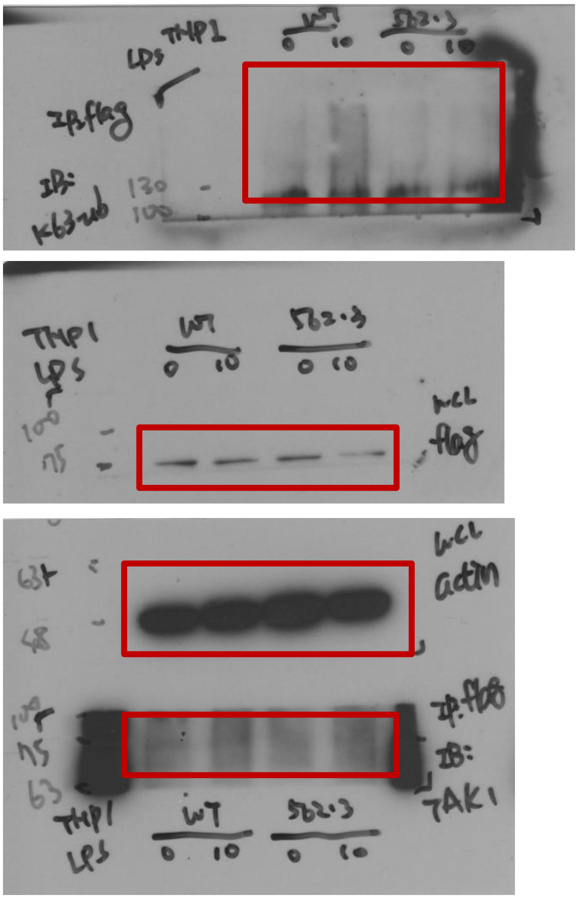

Suppl. Fig5B

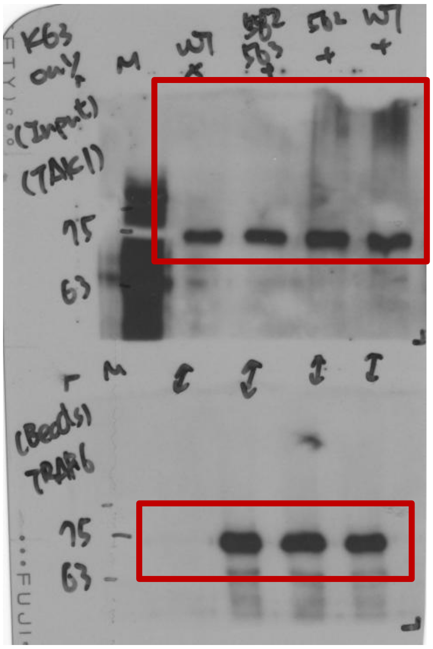

Suppl. Fig5C

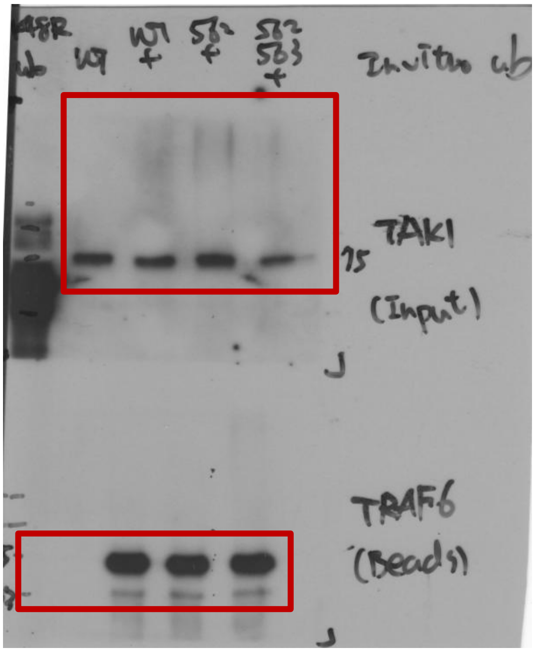

Suppl. Fig5D

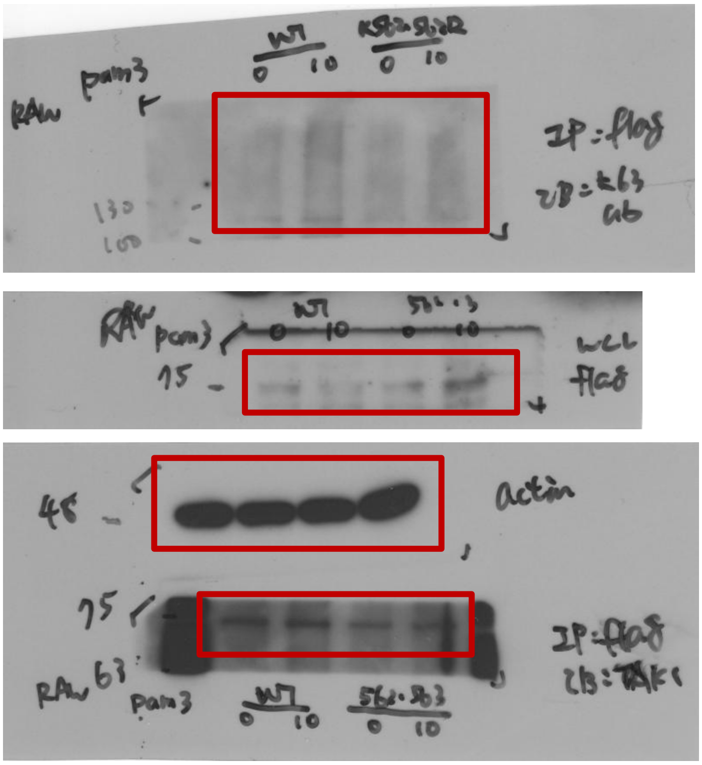

Suppl. Fig6A

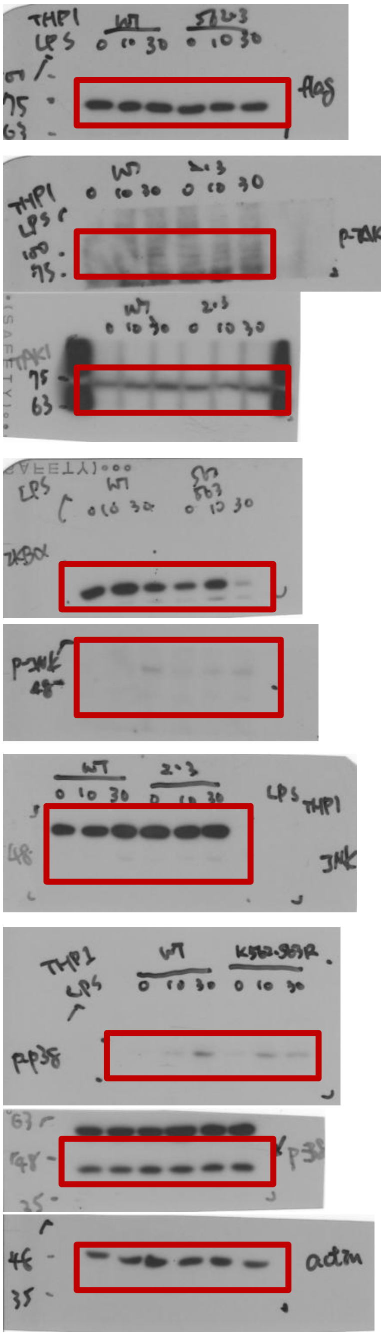

Suppl. Fig6B

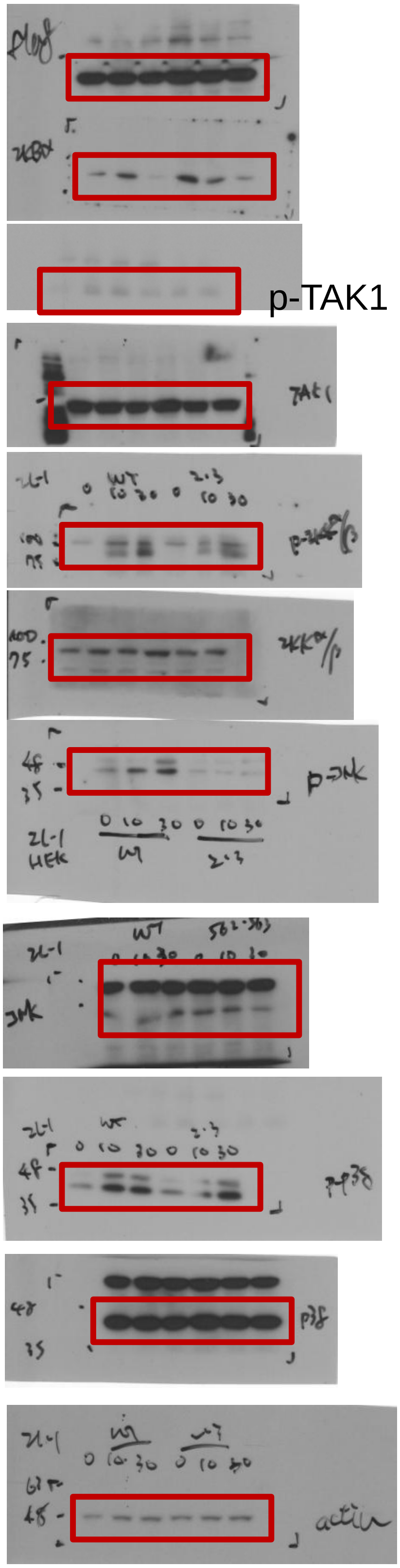

Suppl. Fig6C

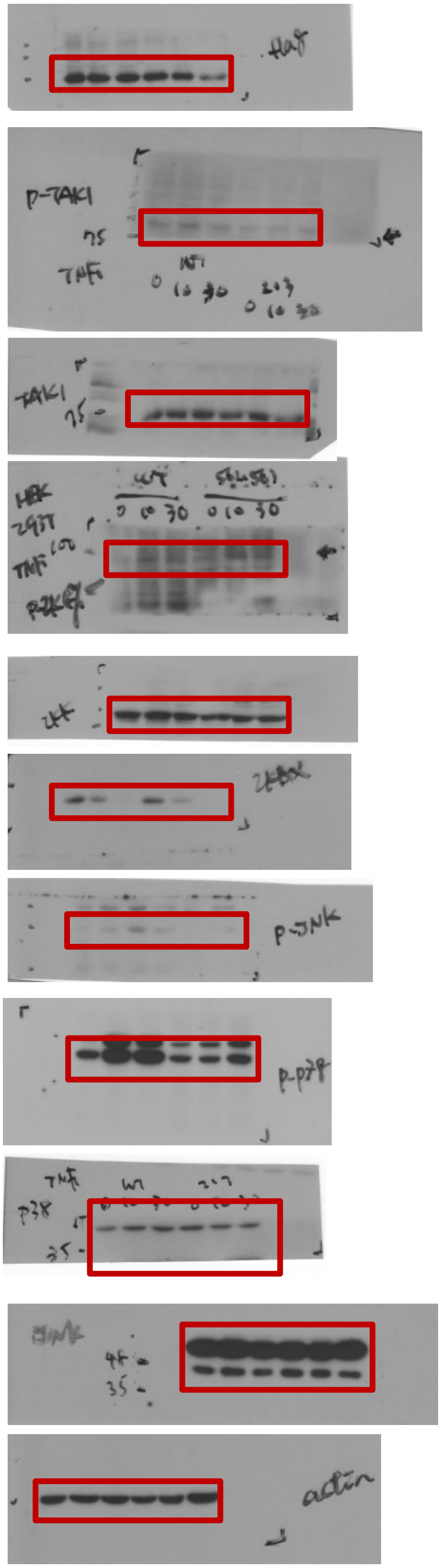

Suppl. Fig7

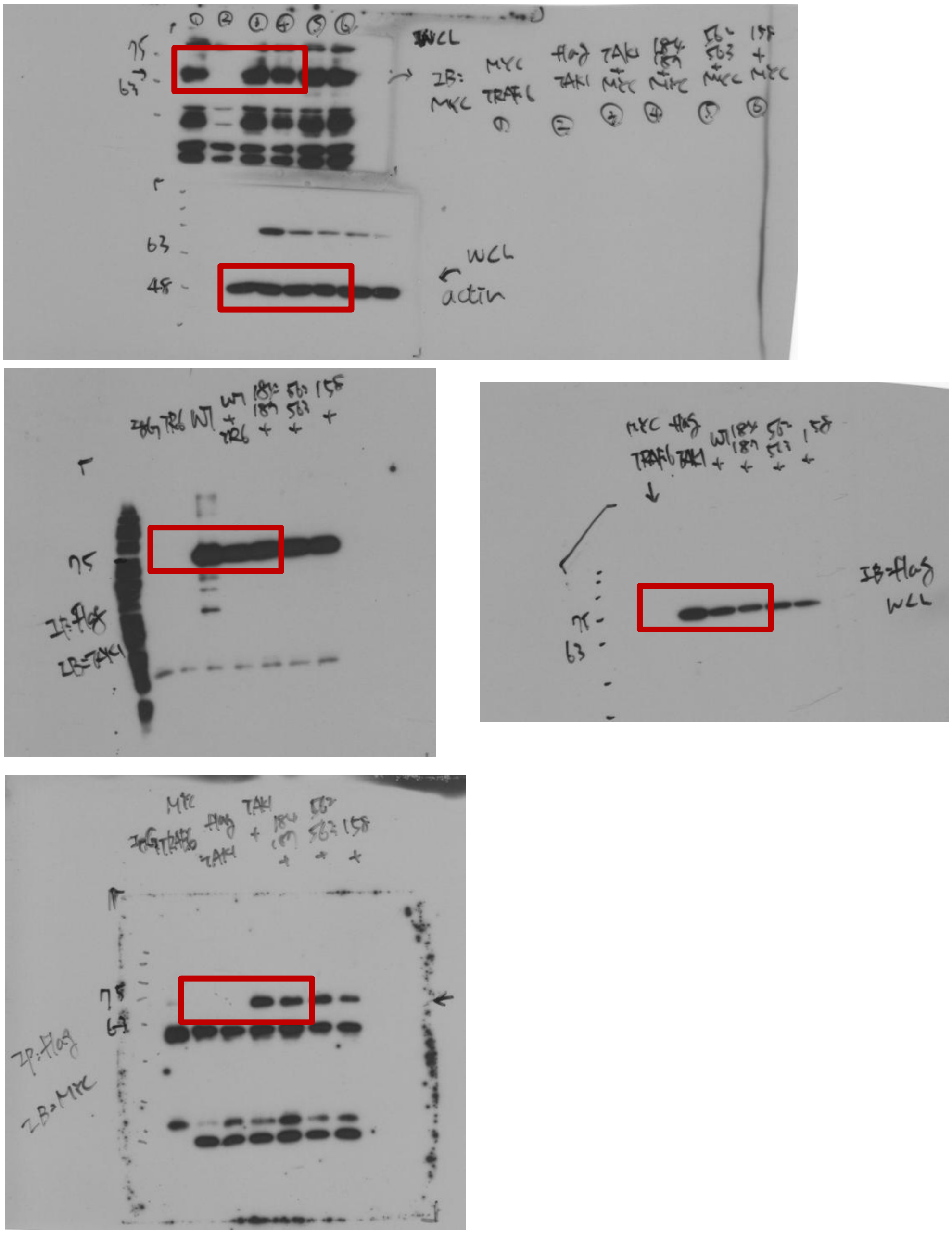

Supplement: Supplementary Information [file srep12300-s1.pdf]
